# Computer vision-derived gait-state tracking of acute treatment response in Parkinson’s disease: external and prospective validation

**DOI:** 10.1038/s41531-026-01614-2

**Published:** 2026-11-11

**Authors:** Florian Lange, Gamze Adaçay, Philipp Köberle, Sai Snigdha Kodali, Ghadir Abbas, Benedikt Weigl, Chi Wang Ip, Muhammad Haseeb, Yann Cordes, Tobias Binder, Muthuraman Muthuraman, Jens Volkmann, Robert Peach, Martin M. Reich

**Affiliations:** 1 Department of Neurology, University Hospital Würzburg, Würzburg, Germany; 2 Institute of Computer Science, University of Augsburg, Augsburg, Germany

**Keywords:** Biomarkers, Computational biology and bioinformatics, Medical research, Neurology, Neuroscience

## Abstract

Continuous, treatment-responsive measurement of motor state is an unmet need in Parkinson’s disease and a broad challenge for digital biomarkers: clinical gait scales are coarse and ordinal, and most digital measures are never validated beyond their derivation setting. We present a digital biomarker derived from full-mesh 3D human-pose reconstruction in standard monocular video, built from high-contrast within-patient treatment transitions and applied to out-of-sample patients. In a retrospective cohort (53 patients) it discriminated treatment states (cross-validated AUROC 0.88) and defined a continuous motor-state axis. Across six independent cohorts (266 subjects, 3,591 walks, eight sites, three continents) it showed consistent medication effects in the two medication-labelled cohorts and tracked clinical gait impairment in the four cohorts carrying clinical gait ratings; in a prospective cohort (21 patients) it preserved ordered transitions for medication and deep brain stimulation. On identical paired observations it was more responsive than the ordinal MDS-UPDRS Part III item 3.10 (standardized response mean 1.29 versus 0.83 internally, 0.92 versus 0.54 externally); its finer resolution also registered directional within-patient improvement more often, an advantage of measurement resolution that reaches parity once change is required to exceed the minimal detectable change, supporting a scalable, generalizable measurement tool.

**Figure:**
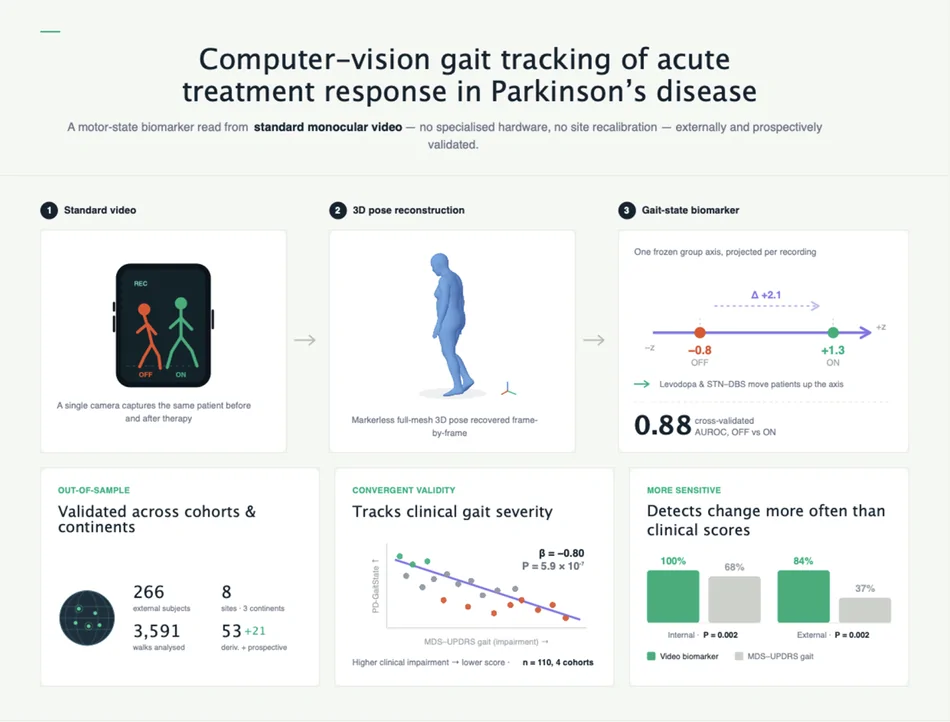

## Introduction

A key need in Parkinson’s disease is to measure motor state continuously and to follow how it responds to treatment. For example, gait impairment is not fixed at a stable level, instead shifting over minutes to hours as dopaminergic medication and deep brain stimulation (DBS) take effect. The clinically informative quantity is therefore the within-patient change a therapy produces, not only the average severity that remains after treatment^1,2^. Current assessment captures only part of this signal. Ordinal scales such as the MDS-UPDRS^3^ are discrete and non-linear, whilst spatiotemporal variables commonly used in gait analysis^4,5^ resolve only a narrow projection of a motor phenotype. Moreover, both ignore coordination, turning, stability, and context-dependent movement organization^3,6,7^. This means that the treatment-responsive component of motor state is captured only coarsely and incompletely, so that real within-patient improvement can fall between the discrete steps of an ordinal scale or be averaged away in a single spatiotemporal summary, and thus there is a critical need to more sensitively measure modifiable motor state.

Video-derived kinematics offer a scalable, low-cost, and objective means of quantifying movement directly from ordinary video, without wearable sensors or specialized motion-capture hardware^8,9^, however, existing video-based approaches generally fail to sensitively and robustly capture motor state for two connected reasons. The first is representational, in that many pipelines rely on 2D pose estimation, including OpenPose-class detectors^10^, which cannot fully capture trunk dynamics, axial coordination, and metric upper-limb movement vigor, all phenotypes that are central to Parkinsonian gait change. The second concerns the target of measurement and the strength of validation. Most video-derived outputs are supervised by and thus trained to reproduce clinical severity scores, which are likely correlated with but not directly tracking patient motor state that is relevant for intervention. Even methods designed for interpretable motor-state tracking^11^ have usually been developed and evaluated within a single setting, with limited evidence from external or prospective deployment^12^. Together, this means that clinically relevant movement structure is only partially measured, and the resulting outputs are rarely tested under the conditions in which a biomarker would need to operate.

To address these existing limitations, we present PD-GaitState, a biomarker whose intended use is to accurately track acute intervention effects in gait. First, we reconstruct full-body motion with markerless, world-grounded 3D estimation using WHAM^13^, yielding temporally resolved kinematics in a metric world frame across axial, head, upper-limb, and lower-limb domains, overcoming the limitations of current 2D video kinematic approaches. Second, rather than explicitly reproducing clinical scores, we derive the biomarker from within-patient treatment transitions, i.e., each patient’s least-treated and dual-ON postoperative recordings (Med-OFF/DBS-OFF versus Med-ON/DBS-ON). This builds on evidence that video-derived kinematics can resolve structured treatment-related change in Parkinson’s disease^14,15^, and anchors the resulting axis to two interventions, dopaminergic therapy and STN-DBS. Third, to make the evidence independent of derivation, the trained model is fixed (no further weight adaptation) before any external or prospective data are evaluated. We then validate PD-GaitState by applying it to (i) a retrospective longitudinal STN-DBS cohort, (ii) six independent external CARE-PD cohorts^16^, and (iii) a prospective cohort. In each, we test whether the video-derived motor-state axis remains a stable and clinically interpretable measure of treatment-responsive gait under dataset shift and real-world deployment.

## Results

### A multi-domain gait-state axis derived from within-patient treatment transitions

We reasoned that the component of motor state most relevant to treatment is the modifiable dysfunction that therapy reverses within a patient, and that this signal is distributed across multiple movement domains. A representation learned to separate a patient’s least-treated and dual-ON states should therefore isolate this treatment-responsive axis more completely than readouts trained on cross-sectional severity or restricted to single variables like walking speed. We tested this by deriving PD-GaitState from high-contrast within-patient transitions, i.e., for each patient, the least-treated postoperative recording (Med-OFF/DBS-OFF) against the dual-ON recording (Med-ON/DBS-ON).

**Fig. 1:**
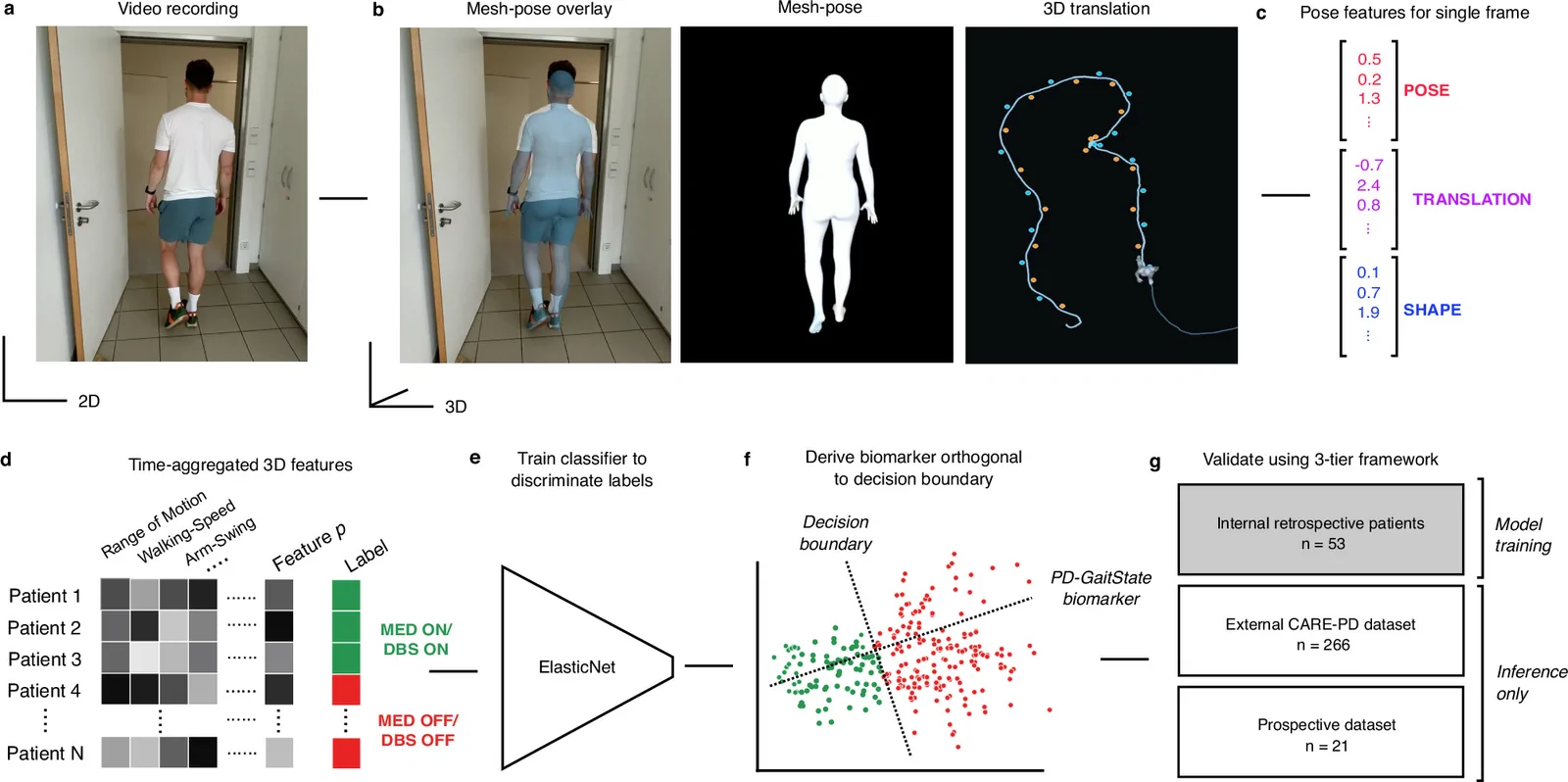
From monocular video to a frozen gait-state biomarker: pipeline and three-stage validation design. An end-to-end measurement chain converts standard single-camera video into a continuous, treatment-responsive motor-state score (PD-GaitState), evaluated along a hierarchical internal → external → prospective ladder. **a** Video recording. Monocular (smartphone-grade) video of overground walking is acquired in a less-treated (OFF) and a more-treated (ON) state. **b** World-grounded 3D reconstruction. Each video is reconstructed with WHAM, yielding a frame-wise mesh-pose overlay, the SMPL body mesh, and the world-grounded 3D root trajectory. **c** Per-frame features. From the SMPL reconstruction, pose, translation and shape parameters are extracted for every frame. **d** Time-aggregated feature matrix. Per-frame signals are aggregated into 168 candidate kinematic features per patient (pose angular velocities and ranges of motion, root-trajectory signals including walking speed, arm-swing, and body-shape parameters); each recording carries a treatment-state label (Med-ON/DBS-ON, green; Med-OFF/DBS-OFF, red). **e** Classifier training. A patient-grouped elastic-net logistic regression discriminates the two extreme treatment states (nested cross-validated AUROC = 0.877 ± 0.067, standard deviation across folds), retaining 24 of 168 features. **f** Biomarker definition. PD-GaitState is the axis orthogonal to the fitted decision boundary; each recording is scored by its signed projection onto this axis, z-standardized over the derivation cohort. **g** Three-tier validation. The complete instrument — features, scaling and coefficients — is frozen after derivation and applied unchanged, with no retraining or recalibration. Stage 1 (internal): derivation in a retrospective STN-DBS cohort (Dataset 1). Stage 2 (external, inference only): transportability in CARE-PD (Dataset 2; six cohorts, 266 subjects, 3591 walks, eight sites), all 24 features computed from externally provided SMPL reconstructions at zero imputation. Stage 3 (prospective, inference only): deployment in a contemporaneous cohort (Dataset 3; 21 patients, 79 runs). Stages 1 and 3 enter at raw video; Stage 2 enters at the SMPL level, testing the downstream biomarker path. **Abbreviations**: STN-DBS subthalamic nucleus deep brain stimulation, Med medication, DBS deep brain stimulation, SMPL Skinned Multi-Person Linear model, WHAM World-grounded Humans with Accurate 3D Motion, AUROC area under the receiver operating characteristic curve, CARE-PD Clinical and AI-Ready Evaluation for Parkinson’s Disease.

**Fig. 2:**
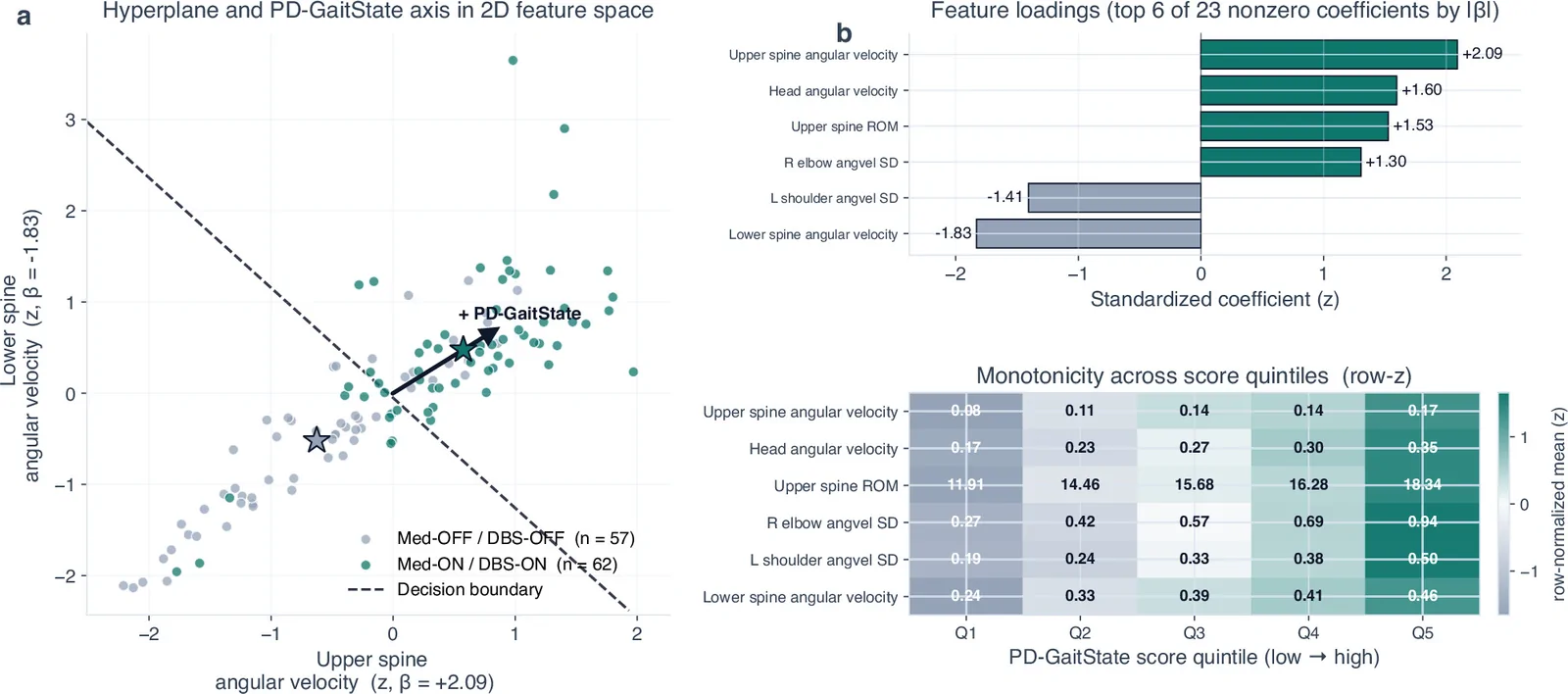
Geometry and feature structure of the frozen PD-GaitState biomarker. **a, Geometric definition**. PD-GaitState is the linear decision function of an elastic-net logistic model fitted in the 24-feature WHAM-derived space to separate Med-OFF/DBS-OFF (less-treated, grey) from Med-ON/DBS-ON (dual-ON, gold) postoperative recordings (Dataset 1; *n* = 119 derivation rows, 53 patients). For visualization the 23 nonzero features are projected onto their two largest-magnitude axes — upper-spine (β = +2.09, x-axis) and lower-spine angular velocity (β = −1.83, y-axis). Stars, class means; dashed line, decision boundary; arrow, unit normal defining the PD-GaitState axis. Each recording’s score is its signed projection onto this axis, z-standardized over the derivation cohort. **b, Feature loadings and monotonic ordering**. Top, standardized coefficients of the six largest-magnitude features (of 23 nonzero; full set provided in Supplementary Table 2); the retained set spans axial, upper-limb and lower-limb domains. Bottom, mean of each feature across PD-GaitState score quintiles (Q1, lowest; Q5, highest), row-z-normalized for visual comparability (raw means inset; *n* = 273 postoperative runs). 22 of 23 features change strictly monotonically across all four quintile transitions (median |*ρ*| = 0.66), a descriptive characterization of score composition indicating that the score traces a continuous, coordinated gradient across its range rather than a discriminative cut. Abbreviations: Med medication, DBS deep brain stimulation, ROM range of motion, ± standard deviation.

From 168 candidate kinematic features computed across axial, upper-limb and lower-limb domains, all derived from world-grounded 3D body motion (WHAM-reconstructed SMPL trajectories), a patient-grouped elastic-net model retained 24 features after correlation pruning, 23 with non-zero coefficients (53 patients, 119 rows). The trained model was then fixed (including feature set, scaling, and coefficients) and then only applied to unseen data in subsequent patient cohorts (Fig. 1). The axis normal to the decision boundary of the trained ElasticNet model then offers a biomarker for each recording (after their projection onto that axis)—from hereon we will refer to this as the PD-GaitState (Fig. 1 for methodological pipeline; Fig. 2a for derived biomarker from real data).

PD-GaitState discriminated the two treatment states well (nested patient-grouped cross-validated AUROC 0.877 ± 0.067; Fig. 2a). To benchmark it fairly against single kinematic features (which carry no tunable hyperparameters and were therefore evaluated on a single-level patient-grouped fold structure, with feature direction fixed within each training fold), we re-scored the composite on that same structure (AUROC 0.847 ± 0.069). On this within-cohort contrast it held no advantage over the strongest individual features (e.g., head angular velocity AUROC 0.861 ± 0.047; right-shoulder angular velocity AUROC 0.849 ± 0.074), each at or above the matched composite; walking speed alone reached AUROC 0.712 ± 0.046. However, this parity does not persist across the full validation ladder: the head and shoulder velocities anchored clinical severity more weakly than the composite in three of the four labelled external cohorts; the upper-spine velocity anchored severity well but did not reach significance for the prospective DBS axis (*P* = 0.091, versus *P* = 3.0 × 10⁻⁵ for the composite); and walking speed, although a strong external severity anchor, was blind to the DBS axis. No individual feature remained strong across every test (Supplementary Table 10).

Two control analyses probed the design choices underlying the axis. First, a severity-supervised counterpart (an elastic net trained on the same features to predict item 3.10) was compared with the treatment-state model head-to-head on a matched basis: both were trained on the same rows (69 recordings from 36 patients carrying item-3.10 ratings) under one shared patient-grouped fold structure, and compared on out-of-fold predictions for the same held-out recordings. The two were statistically indistinguishable on extreme-state discrimination (area under the curve 0.880 versus 0.903; paired difference −0.024, 95% CI [−0.078, +0.035], *P* = 0.45 by patient-level bootstrap), and the severity-supervised model anchored the external UPDRS gait item slightly more strongly in all four labelled cohorts, as expected for a model trained on that rating. We retain the treatment-state target for three reasons: it requires no clinical ratings for training, item 3.10 having been unavailable for a subset of our own visits and the matched comparison itself costing 17 of the 53 derivation patients; it anchors the axis to within-patient physiological interventions, not to rater-dependent labels; and it directly defines the quantity the intended use case tracks (Supplementary Table 6). Second, the frozen score was robust to simulated feature-level measurement noise: injecting Gaussian noise of up to 50% of each feature’s derivation-cohort standard deviation preserved score ranking (median Spearman *ρ* = 0.91 against unperturbed scores), both external medication effects (significant in all 50 replicates per noise level), and the pooled clinical-anchor association (Supplementary Table 7).

### PD-GaitState varies smoothly in its constituent features

The ElasticNet model was trained to separate two extreme motor states of the patient, i.e., no intervention vs. medication and DBS. However, by construction it pays no explicit attention to whether the axis (PD-GaitState) separating those two classes correspond to intermediate motor states. We reasoned that if PD-GaitState indexed a graded motor state, the kinematic features carrying it should change smoothly and monotonically along its length rather than abruptly near the boundary. We tested this on the internal cohort by binning postoperative recordings into PD-GaitState quintiles and tracking each contributing feature across them.

The features traced a smooth gradient (Fig. 2b). Of the 23 contributing features, 22 varied strictly monotonically in their dominant direction across all four quintile transitions, and all 23 were monotonic at three or more transitions; Spearman associations with the score were strong (|*ρ*| ≥ 0.5 for 20 of 23; median |*ρ*| = 0.66). The largest associations lay in the same axial and upper-limb domains that dominated the feature set including head angular velocity (*ρ* = +0.82), upper-spine angular velocity (*ρ* = +0.77), lower-spine angular velocity (*ρ* = +0.69) and upper-spine range of motion (*ρ* = +0.66), each increasing toward the dual-ON end of the axis (Fig. 2b). Movement along PD-GaitState therefore reflects a coordinated, progressive increase in movement amplitude and velocity, so that the score does not behave as a cliff-like cut between two separated clusters. Because the features examined here are constituents of the score, this analysis characterizes score composition descriptively; it does not by itself establish that intermediate positions correspond to intermediate clinical states. That evidence comes instead from the ordered condition means in the prospective cohort and from the independent clinical anchors. PD-GaitState is accordingly read as a treatment-state score computed during walking, whose constituent features change smoothly along its range.

We next asked whether the axis relates to clinical gait constructs beyond item 3.10 in the internal cohort, using condition-matched MDS-UPDRS items from the same postoperative assessments. In subject-clustered condition-level models, PD-GaitState was negatively associated with item 3.11 (freezing of gait; *β* = −0.35 points per z, 95% CI [−0.54, −0.16], *P* = 3.2 × 10⁻⁴), with item 3.12 (postural stability; *β* = −0.65 [ − 0.88, −0.42], *P* = 3.4 × 10⁻⁸) and with an axial composite of items 3.1 and 3.9–3.13 (*β* = −2.22 [−2.85, −1.60], *P* = 2.9 × 10⁻¹²). The complementary levels did not all reach significance for every item: the within-patient change in PD-GaitState between the two extreme treatment states closely tracked the change in item 3.11 (*ρ* = −0.81, *P* = 2.3 × 10⁻⁷, *n* = 28) but not the change in item 3.12 (*ρ* = −0.17, *P* = 0.33), while cross-sectional subject-level means showed the opposite pattern (item 3.12 *ρ* = −0.63, *P* = 2.0 × 10⁻⁶; item 3.11 *ρ* = −0.14, *P* = 0.36), consistent with freezing relating to the treatment-responsive component and postural stability to the trait-like component of the axis. Because levodopa and STN-DBS can affect axial function heterogeneously, we also tested whether high ON-state scores reflected dyskinetic rather than restored movement: ON-state PD-GaitState was not significantly associated with dyskinesia (item 4.1; 46 patients with ratings, 15 with any dyskinesia; *ρ* = 0.19, *P* = 0.22), although modest contributions cannot be excluded (Supplementary Table 8).

This graded structure is a property of the features along the axis and is not contradicted by the signs of the model coefficients, which should not be read as marginal feature directions. Although all 23 retained features were marginally positively associated with the score, 10 carried negative coefficients. Lower-spine angular velocity, for example, took a negative partial weight (*β* = −1.83) despite its positive marginal association, because it is strongly collinear with upper-spine angular velocity (*β* = +2.09; *r* = 0.89) and its coefficient adjusts for shared axial-movement variance (a standard suppressor effect of correlated predictors). Feature-level interpretation therefore rests on the marginal, monotonic associations, a domain-level reading of the axis appropriate to its intended use as a within-patient state measure.

### PD-GaitState registers directional within-patient treatment change more frequently than the ordinal clinical gait rating

A measure intended for treatment tracking must register change within an individual patient, not merely separate groups. The clinical reference, MDS-UPDRS Part III item 3.10 (gait), is limited to five integer levels, meaning that small changes in motor state will be lost. We reasoned that a continuous axis should detect such sub-threshold change more often than the ordinal rating. Because any continuous measure will register more directional changes than an ordinal scale, including changes within measurement noise, we treat this comparison as a test of measurement resolution and complement it below with reliability-derived change thresholds and standardized response means. We tested the detection comparison by comparing (within each patient with paired recordings in two treatment conditions) whether each measure registered improvement, i.e., an increase in PD-GaitState or a decrease in item 3.10, using one-sided McNemar tests.

**Fig. 3:**
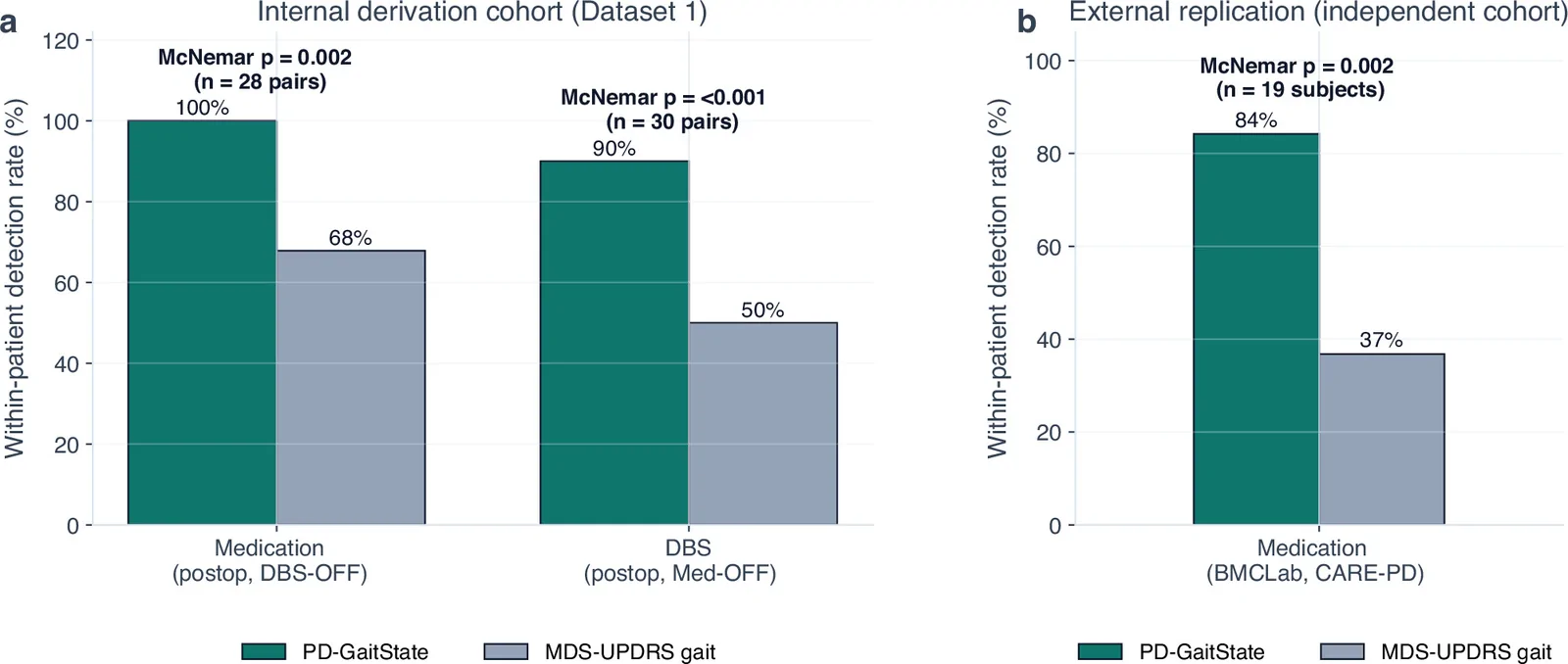
Treatment responsiveness of PD-GaitState versus the clinical gait rating, internal and external. **a Internal derivation cohort** (**Dataset 1**). Bars show the proportion of paired observations in which treatment-related improvement was detected by PD-GaitState (a score increase) versus the MDS-UPDRS gait subscore (item 3.10; a rating decrease), for the postoperative medication contrast under DBS-OFF (*n* = 28 paired observations) and the DBS contrast under Med-OFF (*n* = 30). PD-GaitState detected improvement more frequently in both contrasts (medication, 100% vs 68%, one-sided McNemar *P* = 0.002, 9 vs 0 discordant pairs; DBS, 90% vs 50%, *P* = 0.0009). **b External replication** (**BMCLab, CARE-PD**). The same comparison in an independent cohort with paired Med-OFF/Med-ON recordings and per-walk gait ratings. PD-GaitState detected medication-related improvement in 84% of paired subjects versus 37% for the MDS-UPDRS gait subscore (*n* = 19 subjects; one-sided McNemar *P* = 0.002, 9 vs 0 discordant pairs). Recurrence of the same directional advantage out of sample indicates a stable property of the biomarker and argues against an artefact of the derivation data. Both panels use a zero improvement threshold and therefore contrast the resolutions of the two instruments: at this threshold the continuous score has a split-half false-improvement rate of approximately 50% against approximately 0% for the ordinal rating, and the two converge once improvement is required to exceed the subject-mean minimal detectable change (Supplementary Table 5). Standardized response means on the identical pairs are 1.29 versus 0.83 (internal medication) and 0.92 versus 0.54 (BMCLab). Abbreviations: Med medication, DBS deep brain stimulation, MDS-UPDRS Movement Disorder Society Unified Parkinson’s Disease Rating Scale.

In the internal cohort, PD-GaitState detected improvement more frequently than the clinical rating for both interventions. For the medication contrast under DBS-OFF (28 paired observations), it registered improvement in every pair against 67.9% for the gait subscore (McNemar *P* = 0.002), with all nine discordant pairs favouring PD-GaitState. The DBS contrast under Med-OFF (30 paired observations) showed the same asymmetry, 90.0% against 50.0% (*P* = 0.0009). The advantage here lies in the measurement resolution, wherein both PD-GaitState and clinical scores capture the same direction of treatment effect, but the continuous axis registers the smaller within-patient changes that fall beneath an integer step on the ordinal scale (Fig. 3a).

This property held outside the internal cohort dataset. In BMCLab, an independent cohort carrying both medication labels and per-walk gait ratings for the same subjects, we found the identical comparison reproduced the asymmetry. PD-GaitState detected medication-related improvement in 84.2% of 19 paired subjects against 36.8% for the gait subscore (McNemar *P* = 0.002), again with discordant pairs favouring the biomarker (9 versus 0). That the same directional advantage recurs in an independent cohort suggests it reflects a stable property of PD-GaitState (Fig. 3b).

To quantify responsiveness beyond detection counts, we estimated standardized response means (SRM) for all paired treatment contrasts: internal medication (DBS-OFF) SRM = 1.29 (bootstrap 95% CI 1.03–1.66) and DBS (Med-OFF) SRM = 1.10 (0.82–1.46); external BMCLab medication SRM = 0.92 (0.60–1.36) and E-LC SRM = 0.84 (0.63–1.40); prospective medication SRM = 1.17 (0.75–1.99) and DBS SRM = 1.27 (0.91–1.94). The same statistic computed for item 3.10 on the identical pairs was 0.83 (internal medication) and 0.54 (BMCLab), and the modal BMCLab rating never changed under split-half resampling within unchanged medication state (false-change rate 0%), quantifying the resolution difference from the ordinal side. We further characterized measurement precision from within-state repeat walks in CARE-PD (walk-level standard error of measurement 0.63–1.29 z and walk-level MDC95 1.76–3.58 z across the five state-labelled cohorts, with heterogeneous walk-level ICC(1) of 0.05–0.71; subject-mean MDC95 approximately 0.80 z at the BMCLab median of 20 walks per state) and repeated the detection comparison across improvement thresholds from 0 to 1.0 z. The detection-frequency advantage over item 3.10 persisted for thresholds up to 0.25 z, reached parity once improvement was required to exceed the subject-mean MDC95, and at the strictest threshold (1.0 z) the ordinal rating numerically detected more changes, while split-half analyses within unchanged medication states showed that MDC-thresholded detection controls false-positive change at approximately 1–8% across cohorts (Supplementary Tables 3–5). These walk-level thresholds do not transport directly to the local set-up: a CARE-PD walk is a single walking bout, whereas each local recording aggregates approximately five passes before feature extraction, so the applicable per-recording MDC95 is approximately the walk-level value divided by √5 (0.79–1.60 z), consistent with the two internal same-session repeat recordings (|Δz| = 0.58 and 0.25). The advantage of PD-GaitState therefore lies in resolving sub-integer directional change; a single recording cannot certify small single-condition changes in an individual patient, and both instruments agree once changes are required to exceed their respective measurement errors.

### PD-GaitState tracks clinical severity across independent cohorts with varying acquisition protocols

Our next goal was to validate PD-GaitState with independent data. The frozen model (i.e., all parameters were fixed and we performed inference only) was applied to CARE-PD, a resource of six independent cohorts (266 subjects, 3,591 walks, eight clinical sites in five countries across three continents—Europe, North America and South America) supplying reconstructed 3D body motion.

**Fig. 4:**
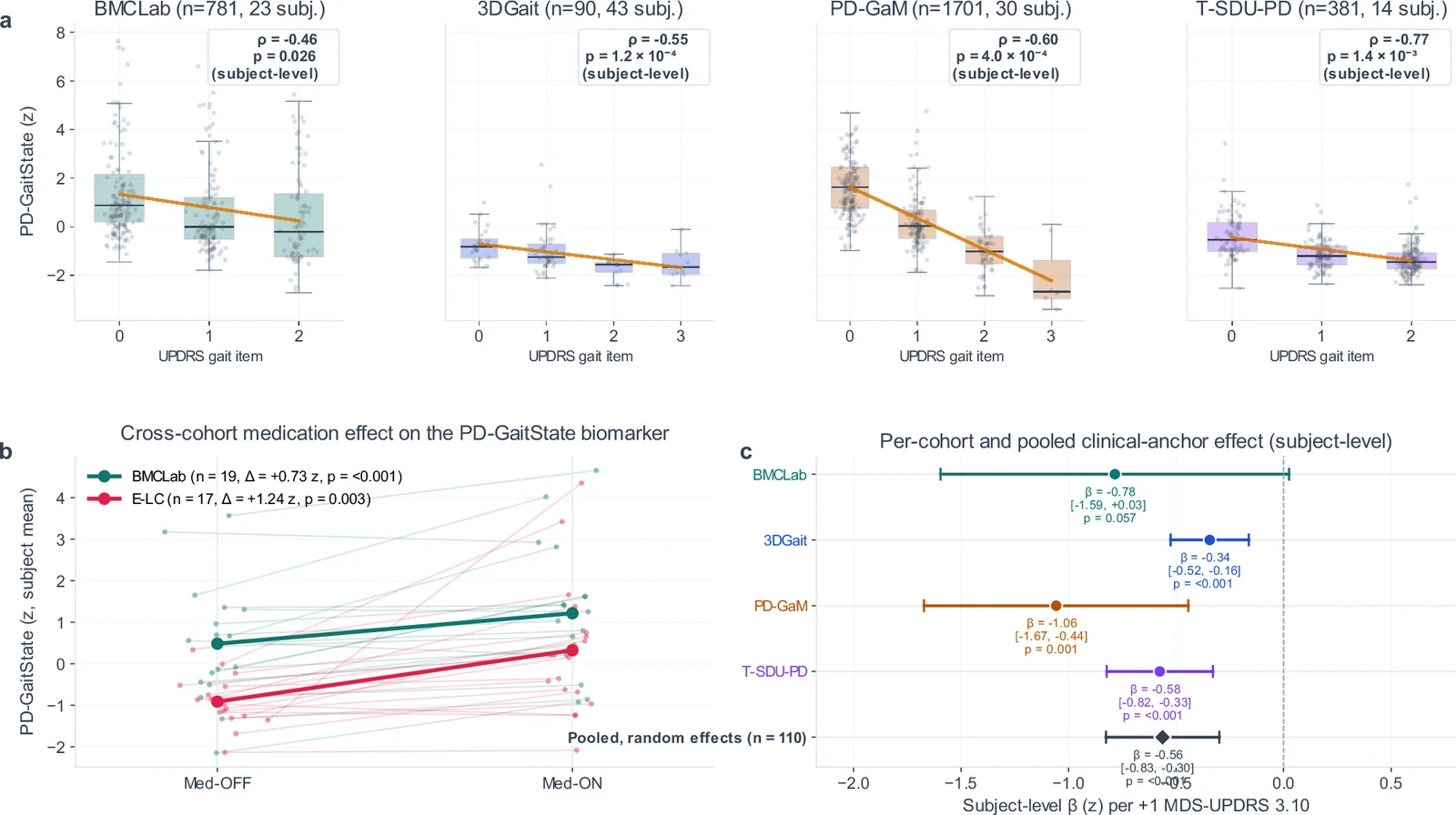
External transportability of the frozen biomarker across six independent CARE-PD cohorts (Dataset 2). **a Clinical anchoring**. Walk-level relationship between the clinician-rated UPDRS gait item (MDS-UPDRS item 3.10 or original UPDRS item 29, depending on cohort) and PD-GaitState (z) in the four CARE-PD cohorts carrying UPDRS-gait labels (BMCLab, 781 walks/23 subjects; 3DGait, 90/43; PD-GaM, 1,701/30; T-SDU-PD, 381/14). Box plots summarize the score distribution at each UPDRS-3.10 level (center line, median; box, 25th–75th percentiles; whiskers, 1.5× interquartile range); box outliers are not drawn separately—instead all walks are overlaid as jittered points (subsampled to ≤350 per cohort for display). Gold line, ordinary-least-squares fit. Insets report the subject-level Spearman ρ (per-subject means) together with its matching subject-level P value. The y-axis is shared across cohorts. **b Treatment-effect transportability**. For each subject in the two medication-labeled cohorts (BMCLab, E-LC) with paired Med-OFF and Med-ON recordings, faint lines connect subject-mean PD-GaitState across states, and the bold line shows the cohort-mean ON–OFF shift. Both cohorts show a significant medication-induced increase (BMCLab, *n* = 19, Δ = +0.73 z, *P* = 0.001; E-LC, *n* = 17, Δ = +1.24 z, *P* = 0.003). **c Pooled clinical anchor**. Forest plot of the subject-level association between PD-GaitState and item 3.10 for each UPDRS-labeled cohort and the pooled estimate (diamond; *n* = 110 subjects). Points, ordinary-least-squares slope on per-subject means (β, z-units per +1-point increase in item 3.10); horizontal lines, 95% confidence intervals. All four cohorts show a negative *β*; the interval excludes zero in 3DGait, PD-GaM, T-SDU-PD, and the pooled estimate (random-effects pooled *β* = −0.56 [−0.83, −0.30], *I*² = 55%, *P* = 2.9 × 10⁻⁵), while BMCLab is directionally consistent (Spearman *P* = 0.026; slope interval marginally includes zero).

We first asked whether PD-GaitState tracked clinical gait impairment. Because walks are heavily nested within subjects and a subject-clustered mixed model did not converge in the high-walk-count cohorts (its random-intercept variance collapsed to the boundary; see “Methods”), we report inference at the subject level, on per-subject means. Across the four cohorts carrying UPDRS-gait labels, higher clinical impairment was associated with lower PD-GaitState in every cohort; a random-effects meta-analysis of the per-cohort subject-level slopes gave a pooled *β* of −0.56 z per point (95% CI − 0.83 to −0.30, *P* = 2.9 × 10⁻⁵; *I*² = 55%; 110 subjects across four cohorts). The association reached significance individually in three cohorts, including PD-GaM^17^ (subject-level Spearman *ρ* = −0.60; OLS slope *β* = −1.06, *P* = 1.5 × 10⁻³; *n* = 30), 3DGait^18^ (*ρ* = −0.55; *β* = −0.34, *P* = 4.4 × 10⁻⁴; *n* = 43) and T-SDU-PD^16^ (*ρ* = −0.77; *β* = −0.58, *P* = 2.7 × 10⁻⁴; *n* = 14), and was directionally consistent in BMCLab^19^, where the rank test was significant but the subject-mean slope marginally included zero (Spearman *ρ* = −0.46, *P* = 0.026; OLS *β* = −0.78 [−1.59, +0.03], *P* = 0.057; *n* = 23). The per-cohort slope magnitude varied roughly threefold (*β* from −0.34 to −1.06), a heterogeneity we attribute to known inter-rater variability in UPDRS-gait rating and to differences in case mix and acquisition across sites (Fig. 4a, c).

We next tested the treatment contrast the biomarker was built to capture. Two cohorts provided paired Med-OFF and Med-ON recordings, and both showed a significant medication-induced increase in PD-GaitState: +0.73 z in BMCLab (*n* = 19, *P* = 0.001) and +1.24 z in E-LC (*n* = 17, *P* = 0.003). The PD-GaitState therefore recovered the direction of dopaminergic improvement in independent unseen cohorts (Fig. 4b).

Finally, the six cohorts together establish that the upstream feature computation generalises. All 24 features were recoverable from independently acquired SMPL reconstructions spanning different cameras, sites and treadmill-versus-overground protocols, without imputation. The pre-trained model produced all features and a well-behaved PD-GaitState distribution with no out-of-distribution collapse (no walk exceeding the |*z*| > 8 reconstruction-artefact threshold).

### Prospective deployment of PD-GaitState recovers both treatment axes beyond individual features

The external cohorts tested generalisation but were unable to test the full pipeline (the pose reconstruction was pre-generated by the respective centers because of data privacy problems of sharing raw patient videos) and moreover did not provide a prospective test of the PD-GaitState. We therefore deployed the full pre-trained pipeline in a prospective evaluation cohort (21 patients, 79 runs) recruited consecutively between August 2025 and April 2026, with the complete pipeline (features, scaling and coefficients) locked in October 2025 before any prospective recording was scored, recorded across the four canonical postoperative conditions (medication off/on × DBS off/on).

**Fig. 5:**
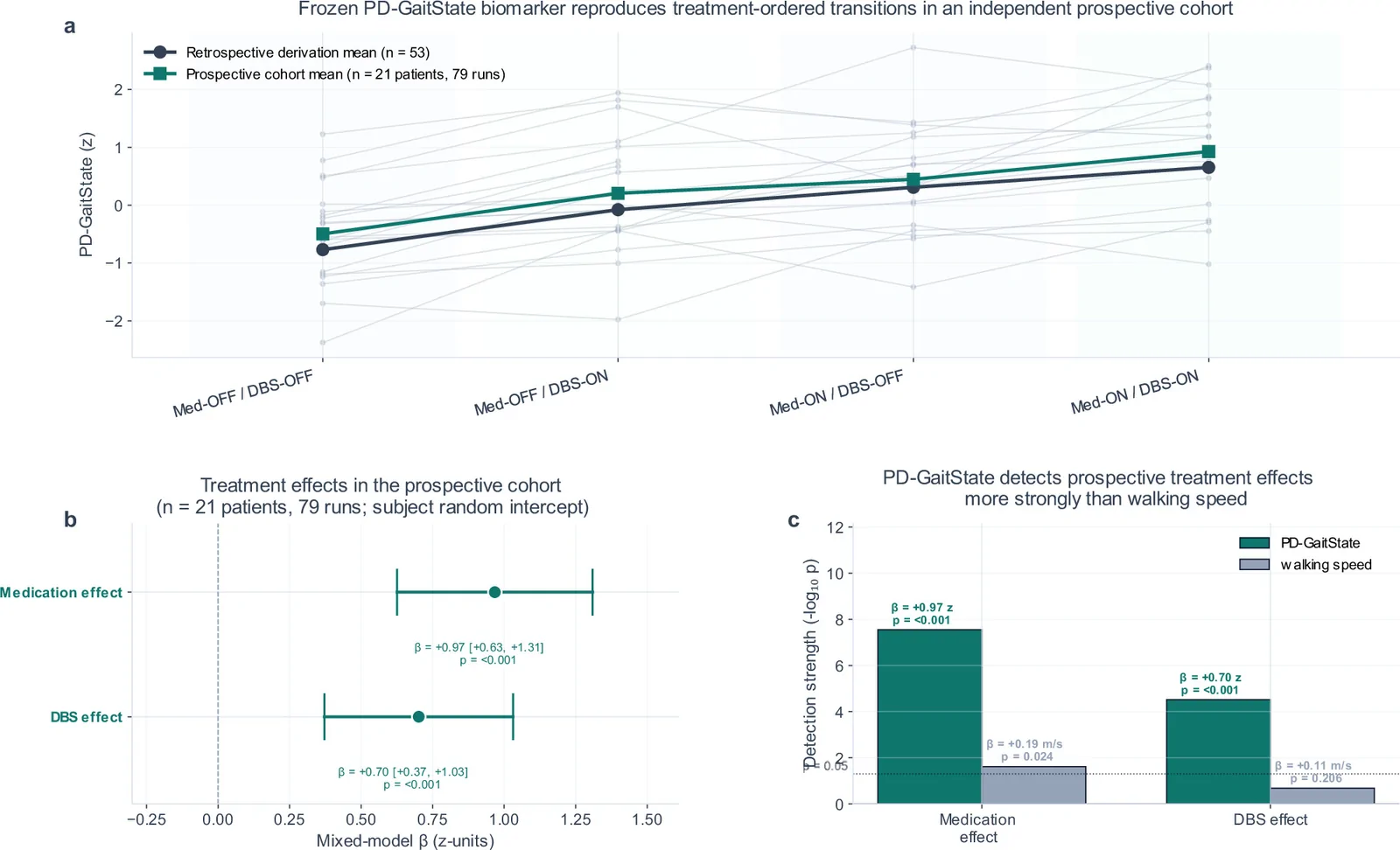
Prospective evaluation: the frozen biomarker reproduces treatment-ordered transitions in an independent cohort (Dataset 3; 21 patients, 79 recordings). **a Treatment-condition trajectory**. Mean PD-GaitState (z) across the four postoperative conditions (Med-OFF/DBS-OFF → Med-OFF/DBS-ON → Med-ON/DBS-OFF → Med-ON/DBS-ON). The biomarker, derived once in the retrospective cohort (navy; Dataset 1, *n* = 53) and never recalibrated, recovers the same ordered trajectory in the prospective cohort (teal; *n* = 21, 79 runs). Faint grey lines, individual prospective trajectories; no error bars are shown. The condition means span 1.4 z (Med-OFF/DBS-OFF, −0.50 z; Med-ON/DBS-ON, +0.93 z). **b, Within-patient treatment effects**. Subject-clustered linear mixed-effects model (gait_state_score_z ~ med_on * dbs_on + (1|patient_id)). Both medication (*β* = +0.97 z, 95% CI [+0.63, +1.31], *P* = 2.8 × 10⁻⁸) and DBS (*β* = +0.70 z, 95% CI [+0.37, +1.03], *P* = 3.0 × 10⁻⁵) produce significant within-patient increases; the medication × DBS interaction was non-significant (*β* = −0.23, *P* = 0.35), consistent with approximately additive effects. Points, fixed-effect estimate; horizontal lines, 95% CI. **c Sensitivity versus walking speed**. Bar height is the statistical detection strength (−log₁₀ *P*) of each treatment effect for PD-GaitState (teal) and walking speed (grey); *β* and *P* are annotated above each bar, and the dotted line marks *P* = 0.05. PD-GaitState detects both effects (medication *P* = 2.8 × 10⁻⁸, DBS *P* = 3.0 × 10⁻⁵), whereas walking speed detects medication only marginally (*β* = +0.19 m s⁻¹, *P* = 0.024) and shows no DBS effect (*β* = +0.11 m s⁻¹, *P* = 0.21), demonstrating that the multivariate axis captures treatment-responsive information beyond speed; the walking-speed medication effect is additionally sensitive to reconstruction quality, whereas neither PD-GaitState effect is (Methods)—most clearly for the DBS axis.

In a per-patient random-intercept model (gait_state_score_z = med_on * dbs_on), both interventions produced significant within-patient increases, medication +0.97 z (95% CI [ + 0.63, +1.31], *P* = 2.8 × 10⁻⁸) and DBS + 0.70 z (95% CI [+0.37, +1.03], *P* = 3.0 × 10⁻⁵), with a non-significant interaction (*β* = −0.23, *P* = 0.35), consistent with approximately additive effects. The four condition means ordered as clinical expectation predicts, from the least-treated state (Med-OFF/DBS-OFF, −0.50 z) through both single-therapy states (Med-OFF/DBS-ON, +0.20 z; Med-ON/DBS-OFF, +0.45 z) to the dual-ON state (Med-ON/DBS-ON, +0.93 z), spanning 1.4 z. The same axis derived once in the retrospective cohort therefore recovered the full treatment ordering in an independent prospective cohort it was never exposed to, with no recalibration (Fig. 5a, b).

Applying the same model to a single variable (walking speed), medication produced an increase (+0.19 m s⁻¹, 95% CI [+0.03, +0.36], *P* = 0.024) whereas DBS did not (+0.11 m s⁻¹, 95% CI [−0.06, +0.27], *P* = 0.21; interaction *P* = 0.44). The medication effect on walking speed was sensitive to specification, ranging from *P* = 0.0015 to *P* = 0.13 across subsets defined by reconstruction quality, while the absence of a DBS effect held in every specification (*P* = 0.22 to 0.44) and both PD-GaitState effects were preserved throughout (see Camera motion, metric scale and the walking-speed comparator). Walking speed therefore recovered the dopaminergic axis only inconsistently and was blind to the stimulation axis, whilst PD-GaitState detected both (Fig. 5c).

## Discussion

The central finding of this study is that a multivariate, video-derived gait-state axis, trained on a retrospective cohort of patients without clinical scores retains directional validity across independent external cohorts (medication effects in the two medication-labelled cohorts, clinical anchoring in the four UPDRS-labelled cohorts, feature recovery in all six) and a prospective evaluation. The primary conceptual contribution is the shift from clinical score modeling to state modeling: the biomarker is anchored to repeated within-patient transitions between less-treated and more-treated conditions. A severity-supervised counterpart transported comparably (Supplementary Table 6), so the case for the state target is operational: it requires no clinical ratings for training, it ties the axis to physiological interventions, and it defines the quantity that treatment tracking must measure. This defines a clinically grounded axis of modifiable dysfunction that is directly tied to physiological intervention and allows for within-patient treatment tracking, necessary for use cases like clinical trials.

Throughout, we accordingly describe PD-GaitState primarily as a treatment-state score computed during walking: what is directly demonstrated is the separation and ordering of treatment states, while its relation to clinically meaningful gait function is established through independent anchors, namely the clinical gait item across four external cohorts and the condition-matched freezing, postural-stability, and axial-item associations in the internal cohort. Because levodopa and STN-DBS can have heterogeneous or even detrimental effects on postural stability, freezing and other axial manifestations, the dual-ON condition need not represent every patient’s best gait state; our dyskinesia analysis found no significant ON-state score inflation in dyskinetic patients, but this construct boundary should be kept in mind when interpreting individual scores.

The McNemar detection analysis provides direct evidence for the measurement-resolution advantage of the continuous axis. The PD-GaitState score registered medication-related improvement in 100% of paired observations compared with 68% for MDS-UPDRS Part III item 3.10, and DBS-related improvement in 90% versus 50%. Importantly, this responsiveness advantage was reproduced in the external cohorts. The consistency of the directional pattern across independent cohorts suggests that this detection-frequency advantage reflects a stable property of the continuous biomarker and not an artifact of the derivation data (Fig. 3). Consistent with a resolution-based account, requiring improvements to exceed the reliability-derived minimal detectable change brought the detection rates of the two instruments to parity, and at the strictest thresholds the ordinal rating numerically detected more, while the standardized response means of PD-GaitState (0.84–1.29 across the primary internal, external and prospective contrasts; 0.47–1.29 when the adjunct contrasts under concurrent therapy are included) show that group-level treatment effects are large relative to its within-state variability. In deployment terms, the reliability analysis becomes a protocol recommendation: each recording already averages several walking passes; the full therapy contrast (1.4 z) exceeds the estimated per-recording MDC95 over most of its range (0.79–1.60 z, internally implied ≈ 1.0 z), while single-therapy effects (+0.70 to +0.97 z) sit at its edge; certifying smaller single-condition changes in an individual patient therefore requires repeat recordings, at the cost of minutes of additional walking. This dissociation reflects the fundamental limitation of an ordinal scale with only five levels (0–4), wherein many patients experience treatment-related gait changes that fall below the threshold for a full integer change in the clinical rating, remaining invisible to the UPDRS while registrable by a continuous measure; individual sub-threshold changes cannot, however, be distinguished from measurement variability without reference to the minimal detectable change. A complementary observation comes from the cross-cohort UPDRS anchor: while the direction of the PD-GaitState–UPDRS association replicated in every labeled cohort, the subject-level slope varied substantially (*β* from −0.34 in 3DGait to −1.06 in PD-GaM, z-units). This magnitude heterogeneity is unlikely to reflect the biomarker–severity relationship alone and is plausibly driven by a combination of well-documented inter-rater variability in MDS-UPDRS gait ratings^20–22^, differences in case mix and UPDRS range sampled, and acquisition protocol differences across sites, sources of variance that a continuous, instrument-defined readout sidesteps while still tracking the same underlying motor signal across cohorts.

The behaviour of walking speed across the validation ladder deserves comment, because it is not uniform. Speed was the strongest single external anchor of the clinical gait item (subject-level *ρ* up to −0.84), which is unsurprising on two counts: gait speed is the most reproducible single parameter of Parkinsonian gait and responds to both dopaminergic therapy and stimulation, and the rating anchors of item 3.10 themselves reference stride amplitude and walking speed, so a speed measure partly reproduces the rater’s primary visual cue. Yet the same variable was the weakest internal discriminator of treatment state (area under the curve 0.712 versus 0.877 for the composite) and did not track the stimulation axis prospectively. Part of this dissociation is a measurement asymmetry: the external cohorts supply reconstructions largely derived from marker-based motion capture, in which translation is metrically precise, whereas in monocular video the same quantity inherits the uncertainty of camera-trajectory and scale recovery. Speed is therefore an efficient proxy for cross-sectional severity wherever it can be measured precisely, but not a sufficient readout of treatment state in the setting this instrument is designed for, and the composite, being rotational, is not exposed to that limitation.

Beyond the fitted score, the study delivers a working measurement chain. Ordinary monocular video (like a smartphone camera or standard video cameras) is carried through to dense, world-grounded 3D body motion, condensed into interpretable kinematic domains, and read out as a frozen gait-state value. Most video work in this area stops at 2D keypoints or a few spatiotemporal summaries^8,10,23,24^; here the video feeds a 3D movement-measurement system that a clinic could in principle run end to end. The chain has one important limit. Our external cohorts entered at the level of 3D body motion, so what transfers across sites is the downstream representation, not the raw-video-to-3D reconstruction step (as raw video cannot easily be shared across centers). Validating that first stage in independent data is the most immediate next step.

The robustness of the axis across cohorts likely reflects the multidimensional structure of Parkinsonian gait impairment. Gait dysfunction spans spatiotemporal, variability, and axial domains^5,25,26^, and motor behavior can be decomposed into partially independent dimensions (including speed, temporal organization, and movement consistency) that are differentially modulated by dopaminergic therapy and DBS^14,15^. A multivariate representation integrating across these domains is less susceptible to cohort-specific variation in any single metric than a readout based on speed or stride length alone. Constructing such a representation depends on recent advances in markerless 3D reconstruction^13^, which provide access to dense kinematic signals (including joint-level angular velocities and multi-segment coordination) that are unavailable from traditional 2D or event-based pipelines. This provides a plausible account of why the biomarker retained directional validity across the CARE-PD cohorts despite dataset shift (medication effects in both medication-labelled cohorts, clinical anchoring in all four UPDRS-labelled cohorts), partial pipeline substitution, and variation across eight independent clinical sites. The CARE-PD pillar also tests the stability of the downstream feature computation itself: identical kinematic features were computed from independently acquired SMPL reconstructions across treadmill versus overground recordings, different cameras, and different sites, and produced a discriminative axis that tracked clinical gait severity in every UPDRS-labelled cohort. This indicates that the downstream representation tolerates substantial acquisition shift. It does not validate the raw-video-to-3D reconstruction step, which each contributing centre performed with its own pipeline; that step remains to be tested in independent data. The approach builds on prior work that has shown video-derived kinematic features capture structured, physiologically meaningful changes under dopaminergic therapy^14,15^ and that markerless systems achieve valid agreement with clinical ratings and gold-standard motion capture^9^. The present work extends this foundation toward generalized biomarker construction under an explicit validation framework.

Interpretability in this context is domain-based. The retained features map onto clinically recognizable constructs (hypokinesia, bradykinesia, and axial dysfunction) consistent with established characterizations of Parkinsonian gait^5,27^. The monotonicity analysis is consistent with this reading: 22 of the 23 contributing features changed strictly monotonically across every score-quintile transition (median |*ρ*| = 0.66). Because the contributing features are constituents of the score, their monotonic behaviour is partly favoured by the correlated structure of the feature space (Supplementary Table 9), and we therefore present this analysis as a descriptive characterization of score composition: a smooth, coordinated gradient from reduced movement at the least-treated end to more vigorous, coordinated gait at the dual-ON end, not an independent demonstration of interval-scale properties. This structural interpretability, grounded in the geometry of the feature space, is appropriate for the intended use case of within-patient state tracking.

Combined, these properties (derivation under high-contrast within-patient transitions, model freezing before any external evaluation, transportability across six independent cohorts, and prospective stress-testing) support the feasibility of the biomarker for its intended use^12^ of within-patient treatment-state tracking under dopaminergic and DBS optimization, with finer measurement resolution for modifiable motor state than the ordinal MDS-UPDRS Part III item 3.10. The present results support feasibility and transportability rather than routine clinical deployment, for which dedicated test–retest characterization, outcome anchoring, and larger prospective cohorts remain necessary. Three concrete features support future deployment: the readout is computed from standard monocular video without specialized capture hardware, the model is applied unchanged to new cohorts without per-site recalibration, and the score is continuous, which supports repeated-measurement protocols that are infeasible with ordinal ratings.

Several limitations define the boundaries of the present claims. The retrospective training cohort remains modest (*N* = 53), and the extreme-state contrast maximizes derivation signal but may limit sensitivity to intermediate or atypical treatment responses. The prospective dataset (*N* = 21) was designed as a deployment-focused evaluation; the sample was pragmatic, and estimates from 21 patients carry corresponding imprecision, which the reported 95% confidence intervals make explicit. External validation in CARE-PD enters the pipeline at the SMPL reconstruction stage, which validates the downstream biomarker path but not the full raw-video sensing chain. The cross-cohort feature set was restricted to pose, root-trajectory, and body-shape signals to ensure complete feature availability across datasets; because these signals are present in all six CARE-PD cohorts, the current validation encompasses all available cohorts (266 subjects, 3591 walks across eight clinical sites). Both local cohorts were also predominantly male (Supplementary Table 1), and we did not evaluate model fairness or performance across sociodemographic subgroups (for example, sex or age); whether PD-GaitState performs equitably across such groups therefore remains to be established. Clinical raters were not blinded to treatment condition; if anything, this favours the clinical rating in the detection comparison, and blinded-rating replication would nonetheless strengthen the finding. Levodopa-equivalent daily dose was not recorded. The walking-speed comparator depends on the reconstructed root trajectory and therefore on monocular scale recovery, which is the one quantity in this study that is not scale-invariant; its treatment effects are reported with the corresponding sensitivity analysis, while PD-GaitState itself is computed from rotational features alone. The reliability estimates derive from within-state repeat walks in the external cohorts rather than a dedicated same-day test–retest protocol in the local set-up, whose protocol yielded one scored recording per condition; repeat-recording test–retest characterization is a planned component of deployment evaluation. The prospective evaluation was not registered, and no formal protocol was prepared; we accordingly describe it as a prospective evaluation rather than a confirmatory validation. In the future, larger prospective cohorts are needed to characterize robustness under routine clinical conditions, and linking the gait-state axis to downstream outcomes (falls, freezing burden, rehabilitation response) would establish clinical utility beyond state tracking while preserving the current claim boundary.

In summary, this work demonstrates that a video-derived gait-state biomarker can be constructed from within-patient treatment transitions, fixed, and shown to retain directional validity across external and prospective cohorts. The geometric ordering of the underlying feature space and the more frequent registration of directional within-patient change relative to the ordinal clinical gait rating indicate that this representation captures a coherent and clinically meaningful motor axis. The results support the feasibility of multivariate, domain-integrated gait representations as interpretable, responsive, and generalizable readouts of modifiable motor dysfunction within a robust validation framework^12^.

## Methods

### Study cohorts and ethics

This study comprised three analytically distinct cohort layers: a Dataset 1 (Internal) derivation set, a prospective clinical validation cohort, and independent external cohorts for transportability testing.

The primary source cohort was a retrospective longitudinal Parkinson’s disease STN-DBS cohort with repeated gait recordings across standard preoperative and postoperative medication and stimulation states. After curation, the dataset included 66 patients and 409 scored runs. Of these, 60 patients contributed complete preoperative medication pairs, and 63 patients (273 runs) had usable postoperative recordings that entered the postoperative mixed-effects analyses. Biomarker derivation additionally required paired extreme postoperative states (both Med-OFF/DBS-OFF and Med-ON/DBS-ON) and predefined analysis-eligibility criteria; the derivation subset therefore comprised 53 patients with 119 runs. Patients were predominantly male (54 of 66), with a mean age of 62 ± 10 years and disease duration of 10 ± 4 years (baseline MDS-UPDRS III 40 ± 12 OFF and 16 ± 9 ON); full per-patient demographic and clinical characteristics are provided in Supplementary Table 1. No formal sample-size calculation was performed for this cohort; all patients with complete gait recordings and informed consent during the study period were included.

Prospective validation used a cohort of 21 patients (79 usable runs) recorded postoperatively across the four canonical bilateral conditions (medication off/on × DBS off/on). All recordings were acquired under identical conditions (see Video acquisition and gait protocol) during a prospective recruitment period from August 2025 to April 2026 (first and last recordings 20 August 2025 and 9 April 2026). Recruitment was consecutive in order of DBS surgery; every approached patient agreed to participate, and no patient was excluded. The complete model and analysis pipeline were locked in October 2025, before any prospective recording was scored or analysed. Twenty-six of the 79 recordings (7 patients) were acquired before the lock; a sensitivity analysis restricted to the 53 post-lock recordings (14 patients) reproduced both effects (medication +1.02 z, 95% CI [+0.58, +1.46], *P* = 6.2 × 10⁻⁶; DBS + 0.56 z, 95% CI [+0.14, +0.99], *P* = 0.010). Recordings were processed with the same pipeline as the retrospective cohort. These patients were likewise predominantly male (18 of 21; mean age 63 ± 12 years; disease duration 10 ± 4 years (available for 12 of 21; one entry corrected from the medical record); Supplementary Table 1). The prospective sample size was determined pragmatically by the number of eligible patients recruited during the deployment window rather than by an a priori power calculation; estimates from 21 patients accordingly carry imprecision, which is reflected in the reported 95% confidence intervals.

External transportability was evaluated using the independent CARE-PD resource^16^, including all six cohorts with compatible pose, root-trajectory, and body-shape signals. This produced 266 subjects and 3591 walks across BMCLab (23 subjects, 781 walks), 3DGait (43 subjects, 90 walks), PD-GaM (30 subjects, 1701 walks), T-SDU-PD (14 subjects, 381 walks), E-LC (59 subjects, 162 walks), and DNE (97 subjects, 476 walks). Four cohorts included UPDRS gait labels (110 subjects, 2953 walks) and two (BMCLab, E-LC) included medication on/off labels (943 walks); these supplied the clinical-anchor and treatment-contrast inference, respectively. The remaining cohort, DNE (97 subjects, 476 walks), carried no clinical or treatment labels and therefore entered no statistical test; it was retained only to assess upstream transportability of the frozen pipeline (feature computability and score-distribution plausibility under acquisition shift), for which all 24 features were computed with zero imputation and no walk exceeded the |*z*| > 8 reconstruction-artefact threshold. As CARE-PD provides reconstructed 3D body motion (SMPL sequences) rather than raw video, this layer evaluates the downstream biomarker pipeline starting from kinematic reconstruction rather than the end-to-end video processing chain.

The project was reviewed by the Medical Ethics Committee of the University of Würzburg, which raised no ethical or legal objections to the research (reference 2026-182-dvhd); all procedures were conducted in accordance with the Declaration of Helsinki. All patients gave written informed consent for video and photographic documentation and for the scientific use of their recordings, including publication, prior to recording; for the retrospective cohort, only recordings from patients who had provided this consent were analysed. The external CARE-PD cohorts were collected under ethics approvals obtained at their respective sites, as detailed in the original data resource^16^.

The study was not registered, and no formal study protocol was prepared. No patients or members of the public were involved in the design, conduct, reporting, or dissemination of this research.

### Video acquisition and gait protocol

All local recordings (retrospective and prospective) were acquired with consumer-grade video cameras (hardware varied over the study period; native frame rate 60 fps) in a standardized acquisition geometry: mounted on a tripod at a height of approximately 1.5 m, approximately 2 m from the walking path at a viewing angle of approximately 45°, without zoom or digital stabilization. Recordings took place in the same gait laboratory under constant lighting, with the full body visible throughout each pass. Patients walked overground along a 5-m path at self-selected speed and without assistive devices; each recording comprised approximately five passes, and turning segments were excluded from analysis. A recording was usable if the patient was fully visible, completed straight-line passes, and the reconstruction passed quality control. Camera intrinsics were not individually calibrated; WHAM’s default intrinsic assumptions were used. Camera-model independence of the downstream biomarker is not assumed but tested: the external validation spans different cameras, sites, and acquisition modalities (Dataset 2), and the noise-perturbation analysis (Supplementary Table 7) shows robustness to feature-level measurement noise.

Postoperative assessments took place at a minimum of three months after surgery. For Med-OFF conditions, dopaminergic medication was withdrawn overnight (≥12 h). For Med-ON conditions, patients received 1.5× their usual morning levodopa dose, and recordings were obtained approximately 60 min after intake. For DBS-OFF conditions, stimulation was switched off with a washout of 45 min before recording. Clinical raters were not blinded to treatment condition. Levodopa-equivalent daily dose was not recorded.

Parkinson’s disease was diagnosed according to the MDS clinical diagnostic criteria, and bilateral STN electrode placement was confirmed by postoperative imaging in all patients. All patients underwent cognitive screening with the Montreal Cognitive Assessment (MoCA) as part of the standard pre-DBS work-up, typically 6–9 months before the three-month follow-up (baseline MoCA available for 54 of 66 retrospective patients: 25.8 ± 2.3; and for 13 of 21 prospective patients: 26.2 ± 2.8). As a retrospectively curated cohort, no formal exclusion criteria were applied; however, no included patient had a history of stroke or debilitating peripheral neuropathy, and patients with severe orthopedic, gait-limiting comorbidity are not offered DBS at our centre as a matter of practice. Freezing of gait, postural stability and dyskinesia were captured through condition-matched MDS-UPDRS items 3.11, 3.12 and 4.1 (see Axial and dyskinesia item analyses). The retrospective and prospective cohorts contained no overlapping participants (verified at the study-identifier level), and the youngest participant (age 26 years) had clinically established young-onset Parkinson’s disease.

### Three-stage validation framework

Validation was organized hierarchically in a V3-inspired digital-biomarker framework^12^. The first stage comprised analytical derivation of PD-GaitState from high-contrast postoperative extreme states plus internal clinical validity in the retrospective cohort. The second stage comprised external transportability testing and claim-boundary definition in independent Dataset 2 (External, CARE-PD). The third stage comprised a final prospective evaluation in Dataset 3 (Prospective). The complete biomarker pipeline was frozen at the end of the internal validation: the selected feature set, imputation and scaling procedure, and fitted model coefficients were then applied unchanged in the external and prospective cohorts, with no feature reselection, recalibration, or coefficient updating.

### Video-derived gait pipeline

For the local retrospective and prospective cohorts, markerless 3D body motion was reconstructed with WHAM^13^, which produces SMPL^28^ pose, translation, and shape trajectories stored as raw output arrays. The use of WHAM-derived SMPL reconstructions enables access to dense 3D kinematic features (including joint-level angular velocities and multi-segment coordination) that are not available from traditional 2D or event-based gait pipelines. Features were extracted directly from these raw WHAM arrays. WHAM was run with its published pretrained checkpoint (wham_vit_bedlam_w_3dpw; ViT backbone trained on BEDLAM with 3DPW fine-tuning), with 2D keypoints from ViTPose-Huge (vitpose-h-multi-coco) and person bounding boxes from YOLOv8x; frame-level pose estimates entered feature extraction without additional smoothing or interpolation, and recordings failing reconstruction quality control were excluded. The cross-cohort feature set comprised 168 candidate features derived from pose-level angular velocities and ranges of motion across body joints, root-trajectory speed, acceleration, and lateral-sway summaries, and body-shape (beta) parameters. This feature set was designed to use only signals present in both local cohorts and external CARE-PD SMPL sequences. This ensures clean cross-cohort transportability without imputation.

### Camera motion, metric scale and the walking-speed comparator

WHAM returns two kinds of output. SMPL pose parameters describe joint rotations in the body frame and are independent of where the camera is and how it moves. The world-grounded root trajectory, by contrast, requires the camera trajectory to be recovered, which WHAM obtains from monocular visual odometry (DPVO); absolute distances additionally depend on the monocular scale, which is not observable from a single uncalibrated view and is resolved by WHAM’s body-scale prior and default intrinsic assumptions.

This distinction matters for interpreting the present results, because all 24 features retained by the frozen model are joint angular velocities and ranges of motion, that is, rotational quantities. PD-GaitState therefore does not use the root trajectory and is invariant to camera motion and to metric scale. Walking speed, used here only as a comparator, is the one reported quantity that depends on both. In the local cohorts, visual odometry was degenerate for a minority of recordings, for which the camera-frame translation was substituted; this substitution was distributed evenly across the four treatment conditions (23 to 26% of recordings per condition, *χ*² *P* = 0.99) and therefore adds variance to the speed comparator without biasing the treatment contrast. Its effect is confined to the trajectory: adjusted for treatment condition, recordings reconstructed from camera-frame translation differed from visual-odometry recordings by +0.53 m s⁻¹ in walking speed (95% CI +0.43 to +0.62) but by +0.07 z in PD-GaitState (95% CI −0.36 to +0.50, *P* = 0.75).

The walking-speed treatment effects were therefore recomputed under alternative specifications: restricted to recordings with a successful visual-odometry trajectory, with speed normalized by reconstructed stature, and after excluding recordings whose reconstructed root path length was implausible for the protocol (> 45 m, against a nominal five passes of a 5-m walkway; six recordings in three patients, with path efficiency 0.02 against a cohort median of 0.10). Across these specifications the medication effect on speed ranged from *P* = 0.0015 to *P* = 0.13, the DBS effect was absent throughout (*P* = 0.22 to 0.44), and both PD-GaitState effects were unchanged (medication *P* ≤ 1.1 × 10⁻⁶, DBS *P* ≤ 3.3 × 10⁻⁴). Those six recordings were retained in all reported analyses: their 24 model features are complete and lie within the derivation-cohort range (maximum |*z*| = 3.7, below the |*z*| > 8 reconstruction-artefact threshold applied to the external cohorts), so the defect is confined to the root trajectory and does not propagate to PD-GaitState.

### Internal validation: analytical derivation of the PD-GaitState biomarker

The PD-GaitState biomarker was derived from features extracted directly from raw WHAM output arrays. The cross-cohort feature set was restricted to 168 features derived from pose angular velocities, joint ranges of motion, root-trajectory kinematics, and body-shape (beta) parameters, all signals available across all cohorts, including external CARE-PD datasets. Derivation rows were restricted to analysis-usable observations. We defined the derivation task as a patient-paired extreme-state discrimination between the least-treated postoperative state (Med-OFF/DBS-OFF) and the dual-ON postoperative state (Med-ON/DBS-ON). Only patients contributing both extreme postoperative conditions entered the derivation set: 53 patients and 119 derivation rows. This extreme-state contrast was chosen intentionally to maximize signal-to-noise during derivation; continuous validity of the resulting axis was then evaluated across intermediate postoperative states, preoperative medication shifts, and external cohorts.

Candidate features were required to have at least 70% observed coverage in the derivation table and to show non-zero variance. The 168 candidate features were not hand-selected for the discrimination task; they enumerate a predefined grid of scalar summaries (means, standard deviations, ranges of motion and related aggregates) of joint angular velocities, joint ranges of motion, root-trajectory kinematics (including walking speed) and SMPL body-shape parameters, computed identically for every recording. Each feature is a scalar, so the feature space is 168-dimensional. Patient identity was never used as a predictor; repeated recordings of the same patient share an identifier only for patient-grouped cross-validation, which prevents leakage between training and test folds. Candidate features were ranked by the absolute standardized mean difference between the two extreme treatment states. The ranked list was correlation-pruned at |*r*| > 0.9, and the final model was limited to 24 retained features.

Selection and hyperparameter tuning were performed strictly within training folds of the nested grouped cross-validation to prevent patient-level leakage. The final held-out test folds were not used for any model selection decisions. Within each training fold, hyperparameters were selected over C ∈ {0.05, 0.1, 0.3, 1.0, 3.0, 10.0} and l1_ratio ∈ {0.1, 0.5, 0.9}. Models were elastic-net-penalized logistic regressions (scikit-learn, saga solver) fitted on median-imputed, z-standardized features; the final refit on the complete derivation table selected C = 3.0 and l1_ratio = 0.5, retaining 23 non-zero coefficients among the 24 selected features. The frozen instrument comprises the feature list, imputation medians, scaling constants, coefficients, and intercept; no component was modified thereafter. This feature-selection strategy was designed to retain domain diversity. The final model was then refit on the complete derivation table. The frozen biomarker was defined as the linear decision function of this model. For interpretability across analyses, raw scores were z-standardized using the derivation mean and standard deviation to produce gait_state_score_z.

The PD-GaitState score is z-standardized on the derivation cohort, so that one score unit corresponds to one standard deviation of the derivation distribution; all treatment effects, β coefficients, and confidence intervals are reported in z-score (standard-deviation) units. This standardization was fixed a priori and does not affect any statistical inference. Interpretability was defined at the level of clinically recognizable gait domains represented in the retained feature set.

### Internal clinical validity

Internal validation of the PD-GaitState biomarker used retrospective runs. Postoperative treatment sensitivity was tested with patient-random-intercept linear mixed-effects models of the form gait_state_score_z ~ med_on * dbs_on. Preoperative medication sensitivity was tested with a paired t-test comparing preoperative Med-ON versus preoperative Med-OFF within patients with both conditions available.

To benchmark the multivariate PD-GaitState axis against simpler gait summaries, we repeated the extreme-state postoperative discrimination task using single metrics under the same patient-grouped fold structure. Single-metric benchmarks included walking speed and each of the 23 contributing model features evaluated individually, scored with ROC AUC, with feature direction set inside each training fold.

### External validation across Dataset 2 (External, CARE-PD)

CARE-PD transportability analysis started from the published dataset. For each of the six included cohorts, individual walks were loaded with their SMPL pose, global translation, shape parameters, frame rate, and available clinical labels (UPDRS gait subscore and/or medication status)^16^. All six cohorts contained the pose, root-trajectory, and body-shape arrays from which all 24 model features could be computed directly, with zero imputation. The same feature extraction as for the retrospective cohort was applied to each CARE-PD walk, and the frozen PD-GaitState model was applied unchanged.

Primary external tests were paired medication comparisons in cohorts with both Med-ON and Med-OFF recordings (BMCLab, E-LC) and per-cohort association between PD-GaitState and the UPDRS gait item. Per the CARE-PD descriptor, the underlying studies rated gait with the MDS-UPDRS gait item (explicitly confirmed for BMCLab and 3DGait) or the original UPDRS gait item; the two rubrics map onto the same severity levels, and we therefore refer to the external ratings generically as the UPDRS gait item. Because walks are nested within subjects (e.g. PD-GaM: 30 subjects, 1701 walks), per-cohort association was summarized at three complementary levels with explicit labeling: (i) a walk-level Spearman rho as a descriptive effect-size summary of the within-cohort association, (ii) a subject-level Spearman rho on per-subject means and (iii) an ordinary least-squares slope on per-subject means (with 95% confidence interval) as the inferential tests. A subject-clustered random-intercept mixed model (gait_state_score_z ~ UPDRS_GAIT) was also examined, but for cohorts with many walks per subject and near-constant within-subject UPDRS-gait labels its random-intercept variance was estimated at the boundary (zero), so the model degenerated toward walk-level pseudoreplication and produced anticonservative p-values; we therefore report the convergence-independent subject-level statistics as the primary inference. Inferential p-values reported in Results are subject-level (per-subject-mean) statistics. The pooled cross-cohort anchor was estimated by DerSimonian–Laird random-effects meta-analysis of the per-cohort subject-level OLS slopes, with heterogeneity summarized by *I*²; a single regression pooled across cohorts was avoided because it absorbs between-cohort differences in severity and score level; per-cohort trend lines in Fig. 4a are descriptive within-cohort fits; inference is from the subject-level OLS slope on per-subject means, summarized as a forest plot in Fig. 4c. Because BMCLab provided both medication labels and per-walk UPDRS gait ratings for the same subjects, we also replicated the McNemar responsiveness comparison in this external cohort using the same framework as the internal analysis. For each subject with paired medication recordings, subject-level means of gait_state_score_z and modal UPDRS gait ratings were computed separately for the Med-OFF and Med-ON conditions. Improvement was defined as an increase in gait_state_score_z or a decrease in the modal UPDRS gait rating, and detection rates were compared with a one-sided McNemar exact test (binomial on discordant pairs). Stage 2 (External validation) thus tested the downstream biomarker path under dataset shift.

### Prospective validation in a Dataset 3 (Prospective): a contemporaneous cohort

The Dataset 3 (Prospective) was designed to test whether the frozen biomarker behaved correctly under real-world deployment constraints. Primary prospective validation consisted of postoperative linear mixed-effects models on the externally scored PD-GaitState using the same fixed-effects structure as in the retrospective postoperative analysis, gait_state_score_z ~ med_on * dbs_on, with a patient random intercept. Condition-wise summaries and separate raw within-patient transition tables were then computed for the three canonical treatment actions: medication on, DBS on, and combined medication-plus-DBS on. Model fitting used maximum likelihood with the lbfgs optimizer, and convergence was confirmed before reporting the prospective fixed-effect estimates.

### Statistics

All analyses were performed in Python. Mixed-effects models were fit with random intercepts for patient and fixed effects for medication, DBS, and their interaction. Paired within-patient contrasts were tested with paired two-sided t-tests and are reported with 95% confidence intervals; standardized response means (mean paired difference divided by the standard deviation of the paired differences) are reported with bootstrap 95% confidence intervals (5000 resamples). Monotonic external associations were summarized with Spearman’s rho. We did not apply explicit algorithmic fairness or bias-mitigation procedures; PD-GaitState is computed from objective kinematic features and does not use protected attributes (for example, sex, age or ethnicity) as inputs. Model performance across sociodemographic subgroups was not formally evaluated, as subgroup sizes were small and the external cohorts did not provide harmonised demographic metadata.

Cross-validation was grouped at the patient level to avoid leakage between repeated measurements from the same individual. The primary biomarker derivation used nested patient-grouped GroupKFold cross-validation with up to 5 outer folds. Missing numeric values in model pipelines were handled with median imputation followed by standard scaling. The main discrimination metric for the frozen PD-GaitState derivation was area under the receiver operating characteristic curve.

### Monotonicity analysis

To assess whether the PD-GaitState biomarker defined a geometrically ordered motor gradient, we tested the monotonicity of each contributing feature across score quintiles. Retrospective postoperative usable runs were binned into five equally sized groups by gait_state_score_z. For each of the 23 contributing features, we computed (i) the Spearman rank correlation with the continuous score and (ii) a monotonicity score defined as the fraction of adjacent quintile transitions in which the feature mean moved in the dominant direction (i.e., 1.0 if all four transitions were consistently increasing or decreasing). Features were classified as strictly monotonic if all adjacent quintile means changed in the same direction.

To contextualize the monotonicity count, two null references were computed on the same runs and with the same criterion: random unit axes in the standardized feature space (1,000 replicates) and axes from elastic nets fitted, with identical hyperparameters, to treatment labels permuted within patient (200 replicates); results are reported in Supplementary Table 9.

### Test–retest reliability and minimal detectable change

Within-state repeat walks in CARE-PD provided the repeat structure for reliability estimation, with state defined by the finest available labels: subject × medication state × gait rating in BMCLab, subject × medication state in E-LC, and subject × gait rating in the per-walk-rated cohorts. DNE carries no state label and is reported only as an upper bound on measurement error, because residual state variation remains in its error term. One-way random-effects variance components yielded the intraclass correlation coefficient ICC(1), the standard error of measurement (SEM; within-group standard deviation) and the walk-level minimal detectable change (MDC95 = 1.96 × √2 × SEM); the subject-mean MDC95 divides the SEM by √k for k walks per state. The internal protocol yielded one scored recording per condition, so a dedicated internal test–retest estimate was not available. As sensitivity analyses, the McNemar comparison was repeated with improvement thresholds between 0 and 1.0 z and at the subject-mean MDC95, and split-half analyses within unchanged medication states estimated false-positive change rates at each threshold. Because each local recording aggregates approximately five walking passes before feature extraction, whereas CARE-PD scores refer to single walking bouts, per-recording measurement error was approximated as the walk-level SEM divided by √5, giving an estimated per-recording MDC95 of 0.79–1.60 z across the state-labelled cohorts; the two available internal same-session repeat recordings (|Δz| = 0.58 and 0.25, implying a per-recording SEM near 0.37 z) are consistent with this range. The transport rests on two approximations (equal per-pass noise across settings and independent passes), and a dedicated repeat-recording protocol remains the definitive test.

### Severity-supervised benchmark

To test whether supervision by clinical severity would outperform treatment-state supervision, an elastic-net linear regression predicting item 3.10 was trained on the same 24 features under identical patient-grouped nested cross-validation, frozen, and applied to the external and prospective cohorts with the same procedures as PD-GaitState.

### Noise-perturbation analysis

Robustness to feature-level measurement noise was assessed by re-implementing the frozen scorer from its persisted coefficients and scaling constants (validated against stored scores; Pearson *r* = 1.000) and injecting zero-mean Gaussian noise with standard deviation equal to 5–50% of each feature’s derivation-cohort standard deviation into the external feature table (50 replicates per level), tracking score rank stability, the paired external medication effects and the pooled clinical-anchor association. The injected noise is independent and identically distributed across features and walks; it does not reproduce the structured, pose-dependent error of monocular reconstruction and is best read as a first-order robustness probe.

### Axial and dyskinesia item analyses

Condition-matched MDS-UPDRS items 3.11 and 3.12 and an axial composite (items 3.1 and 3.9–3.13, where available) were extracted from the clinical database using the same visit-condition mapping as item 3.10 (verified empirically with 100% agreement against the item 3.10 lookup). Associations with PD-GaitState were summarized at condition level, at subject level (per-subject means) and as within-patient changes between the two extreme postoperative states. ON-state dyskinesia (item 4.1) was tested for association with ON-state PD-GaitState (Spearman correlation and dyskinetic versus non-dyskinetic group comparison).

### Responsiveness comparison with clinical gait ratings

To compare the within-patient responsiveness of PD-GaitState against the ordinal MDS-UPDRS item 3.10 gait subscore, we identified retrospective patients with matched recordings and UPDRS_GAIT assessments in both the less-treated and more-treated conditions. For each paired observation, we classified whether each measure detected improvement: an increase in gait_state_score_z (indicating a shift toward the dual-ON end of the axis) or a decrease in the item 3.10 score (indicating clinical improvement). Detection rates were compared with one-sided McNemar exact tests (binomial test on discordant pairs, testing the alternative hypothesis that PD-GaitState detected improvement more frequently than UPDRS-gait). Analyses were performed separately for medication contrasts (paired across DBS states) and DBS contrasts (paired across medication states) and pooled across the concurrent treatment condition.

## Supplementary information

Supplementary Information

## Data Availability

Raw video recordings cannot be shared owing to data-protection constraints. The downstream analytic data supporting the findings of this study are available from the corresponding author on reasonable request.

## Code availability

The custom code used to derive the biomarker and generate the results of this study is available from the corresponding author on reasonable request.
