# Supplementary material for: Computer vision-derived gait-state tracking of acute treatment response in Parkinson’s disease: external and prospective validation
Source: NPJ Parkinsons Dis. 2026 Nov 11;12:279. doi: 10.1038/s41531-026-01614-2 (PMC13755425; doi:10.1038/s41531-026-01614-2)
Supplement: Supplementary file 1 [file 41531_2026_1614_MOESM1_ESM.pdf]

Supplementary Information

Computer vision-derived gait-state tracking of acute treatment response in Parkinson's disease: external and prospective validation

Lange et al.

Contents

Supplementary Table 1 | Per-patient demographic and clinical characteristics (retrospective and prospective cohorts)

Supplementary Table 2 | PD-GaitState model coefficients (24 selected features)

Supplementary Table 3 | Within-state reliability of PD-GaitState in CARE-PD (walk level, state-aware units)

Supplementary Table 4 | Standardized response means (SRM) with 95% CIs for all paired treatment contrasts

Supplementary Table 5 | Detection-threshold sensitivity analysis of the McNemar comparison and split-half specificity

Supplementary Table 6 | Matched head-to-head comparison of treatment-state and severity supervision

Supplementary Table 7 | Robustness of the frozen score to feature-level Gaussian noise

Supplementary Table 8 | Associations of PD-GaitState with axial MDS-UPDRS items and dyskinesia

Supplementary Table 9 | Null references for the feature-monotonicity count

Supplementary Table 10 | Single features versus the composite across the full validation ladder

Supplementary Table 1. Patient-level demographic and clinical characteristics

Per-patient listing for the internal (Dataset 1) and prospective (Dataset 3) cohorts of the PD-GaitState study. Demographic and clinical values are baseline measurements drawn from the institutional clinical database; recording counts are from the frozen-model scoring tables. MDS-UPDRS III and gait item 3.10 are reported in the medication-OFF and medication-ON states at baseline. Patient identifiers (R##, P##) are anonymized study codes; the key linking them to source records is held separately by the study team. The external CARE-PD cohorts (Dataset 2) are not listed here; their demographics are described in the source resource (Adeli et al., 2025).

Retrospective cohort (Dataset 1 — internal derivation/validation)

Patient ID	Set	Sex	Age (yr)	Disease dur. (yr)	BMI (kg/m²)	MDS-UPDRS III OFF	MDS-UPDRS III ON	Item 3.10 OFF	Item 3.10 ON	Recordings (n)	Conditions (n)
R01	Derivation	M	70	—	—	—	—	—	—	5	5
R02	Derivation	F	69	4	28.3	47	11	2	1	9	6
R03	Derivation	F	70	9	26.2	41	23	2	2	6	6
R04	Derivation	M	47	5	26.1	30	5	1	0	6	6
R05	Derivation	F	70	9	—	33	13	2	1	6	6
R06	Derivation	M	65	12	28.1	42	12	2	1	6	6
R07	Derivation	M	48	6	29.4	38	4	1	0	6	6
R08	Derivation	M	70	10	25.2	42	21	1	1	6	6
R09	Validation	M	68	15	29.1	30	16	2	1	5	4
R10	Derivation	F	63	12	25.2	60	21	2	1	8	6
R11	Validation	M	64	18	22.6	33	12	2	1	2	2
R12	Derivation	M	75	15	25.6	46	18	2	1	5	5
R13	Derivation	M	72	10	21.8	41	14	1	1	6	6
R14	Derivation	M	69	7	25.5	37	11	1	0	7	6
R15	Derivation	M	69	26	—	—	—	—	—	6	4
R16	Derivation	F	62	15	21.9	—	—	—	—	7	6
R17	Derivation	M	62	5	26.9	20	2	0	0	8	6
R18	Validation	M	40	4	25.6	46	11	3	1	6	4
R19	Validation	F	71	16	21.5	40	21	2	1	7	3
R20	Derivation	—	—	—	—	—	—	—	—	6	6
R21	Derivation	F	59	7	19.8	63	13	2	0	6	6
R22	Validation	M	49	5	21.9	—	—	—	—	3	3
R23	Validation	M	78	13	—	43	17	2	1	7	5
R24	Validation	M	65	9	—	—	—	—	—	6	5
R25	Validation	M	69	8	21.7	40	40	2	2	3	3
R26	Validation	M	73	15	27.4	42	34	4	4	4	4
R27	Derivation	M	50	11	32.1	61	47	2	1	5	5
R28	Validation	M	58	8	28.1	27	19	1	1	8	3
R29	Validation	M	75	10	22.8	17	8	1	0	2	2

Patient ID	Set	Sex	Age (yr)	Disease dur. (yr)	BMI (kg/m²)	MDS-UPDRS III OFF	MDS-UPDRS III ON	Item 3.10 OFF	Item 3.10 ON	Recordings (n)	Conditions (n)
R30	Derivation	M	37	6	25.5	30	14	1	1	10	6
R31	Derivation	M	65	9	—	—	—	—	—	6	6
R32	Derivation	M	70	11	30.0	42	41	2	2	6	6
R33	Derivation	M	55	8	—	62	12	2	1	6	6
R34	Derivation	F	26	4	—	30	8	0	0	6	6
R35	Derivation	M	63	9	23.1	42	14	2	1	6	6
R36	Derivation	M	62	12	23.8	55	21	2	1	6	6
R37	Validation	M	70	16	—	—	—	—	—	7	5
R38	Validation	M	67	12	25.6	35	17	2	1	4	2
R39	Derivation	M	51	10	23.1	—	—	—	—	5	5
R40	Derivation	M	56	14	21.8	33	16	1	0	6	6
R41	Derivation	M	72	4	29.0	39	21	1	1	6	6
R42	Derivation	M	63	13	24.6	33	17	2	1	6	6
R43	Derivation	M	53	12	23.9	40	16	1	0	6	6
R44	Derivation	M	63	16	—	20	7	1	0	6	6
R45	Derivation	M	58	11	19.6	57	16	2	1	8	6
R46	Derivation	M	75	14	27.2	—	—	—	—	6	6
R47	Derivation	M	59	11	24.8	42	25	2	2	8	6
R48	Derivation	M	50	5	—	24	12	2	1	6	6
R49	Derivation	M	62	12	28.1	48	23	3	0	11	6
R50	Derivation	M	56	8	—	27	8	1	1	6	6
R51	Derivation	F	54	11	19.2	24	8	2	0	6	6
R52	Derivation	M	49	3	30.9	32	7	0	0	6	6
R53	Derivation	M	65	13	39.1	37	20	2	1	10	6
R54	Derivation	F	67	11	28.8	28	16	2	1	6	6
R55	Derivation	M	66	14	29.6	51	16	2	1	6	6
R56	Derivation	M	66	14	24.8	41	13	2	1	6	6
R57	Derivation	M	—	—	—	—	—	—	—	6	6
R58	Derivation	M	67	7	17.9	42	17	2	1	6	6
R59	Derivation	F	71	9	24.4	37	19	2	2	6	6
R60	Derivation	M	60	6	24.1	65	24	1	0	8	6
R61	Derivation	M	48	5	23.1	36	10	2	0	6	6
R62	Derivation	M	65	9	34.6	40	17	1	0	9	6
R63	Derivation	M	62	22	—	32	17	1	1	6	6
R64	Derivation	M	71	14	24.5	64	15	2	1	6	6
R65	Derivation	M	60	—	—	—	—	—	—	6	6

Patient ID	Set	Sex	Age (yr)	Disease dur. (yr)	BMI (kg/m²)	MDS-UPDRS III OFF	MDS-UPDRS III ON	Item 3.10 OFF	Item 3.10 ON	Recordings (n)	Conditions (n)
R66	Derivation	M	74	11	26.3	28	9	2	0	6	6
**Summary (mean ± SD)**	**n = 66**	**54M / 11F**	**62 ± 10**	**10 ± 4**	**25.6 ± 3.9**	**40 ± 12**	**16 ± 9**	**1.7 ± 0.7**	**0.8 ± 0.7**	**409**	

Prospective cohort (Dataset 3 — contemporaneous deployment)

Patient ID	Set	Sex	Age (yr)	Disease dur. (yr)	BMI (kg/m²)	MDS-UPDRS III OFF	MDS-UPDRS III ON	Item 3.10 OFF	Item 3.10 ON	Recordings (n)	Conditions (n)
P01	Prospective	M	66	14	31.2	65	31	4	2	4	4
P02	Prospective	M	67	11	—	48	18	2	1	4	4
P03	Prospective	F	57	17	28.3	35	8	1	0	4	4
P04	Prospective	M	71	16	—	19	11	1	1	4	4
P05	Prospective	M	61	12	—	67	22	3	1	4	4
P06	Prospective	F	67	13	—	62	16	3	0	4	4
P07	Prospective	M	71	8	—	45	31	2	2	4	4
P08	Prospective	M	59	10	—	36	14	2	1	3	3
P09	Prospective	M	79	14	—	—	—	—	—	4	4
P10	Prospective	M	63	13	—	—	—	—	—	4	4
P11	Prospective	F	27	5	—	—	—	—	—	4	4
P12	Prospective	M	67	15	—	—	—	—	—	4	4
P13	Prospective	M	71	12	—	—	—	—	—	2	2
P14	Prospective	M	67	19	—	—	—	—	—	4	4
P15	Prospective	M	45	15	—	—	—	—	—	4	4
P16	Prospective	M	62	13	—	—	—	—	—	4	4
P17	Prospective	M	57	8	—	61	35	2	2	4	4
P18	Prospective	M	—	—	—	33	13	2	1	4	4
P19	Prospective	M	74	5	—	31	14	2	2	4	4
P20	Prospective	M	51	6	—	—	—	—	—	4	4
P21	Prospective	M	70	5	26.8	49	12	2	1	2	2
**Summary (mean ± SD)**	**n = 21**	**18M / 3F**	**63 ± 12**	**10 ± 4**	**28.8 ± 2.2**	**46 ± 15**	**19 ± 9**	**2.2 ± 0.8**	**1.2 ± 0.7**	**79**	

Footnotes

Patient ID: anonymized study identifier (R## retrospective, P## prospective). Identifiers do not encode any clinical or recruitment information.

Set: role of each patient in the study. Derivation, used to fit the frozen PD-GaitState model (53 retrospective patients, 119 paired extreme-state recordings); Validation, retrospective patients used for internal validation only and held out from model fitting; Prospective, contemporaneous deployment cohort (Dataset 3).

Age, disease duration, BMI, MDS-UPDRS III and item 3.10: baseline visit values. Age computed from year of baseline visit minus birth year. BMI = weight / height². Dashes (—) indicate values not available.

Recordings / Conditions: number of usable gait recordings and distinct treatment conditions per patient (totals: 409 retrospective, 79 prospective).

Sex: M, male; F, female.

Supplementary Table 2 | PD-GaitState model coefficients (24 selected features)

Feature	Standardized coefficient
pose_spine3_angvel_mean	2.0908
pose_spine1_angvel_mean	-1.8306
pose_head_angvel_mean	1.5967
pose_spine3_rom_deg	1.5263
pose_left_shoulder_angvel_std	-1.4063
pose_right_elbow_angvel_std	1.3044
pose_neck_angvel_std	1.2571
pose_spine2_rom_deg	-0.9583
pose_left_shoulder_angvel_mean	0.6558
pose_all_angvel_mean_iqr	-0.4099
pose_left_ankle_rom_deg	0.4014
pose_right_shoulder_angvel_mean	0.3986
pose_right_foot_rom_deg	0.3948
pose_all_rom_deg_median	-0.3724
pose_left_wrist_angvel_mean	-0.305
pose_right_shoulder_angvel_std	-0.2812
pose_all_angvel_mean_std	0.2721
pose_left_foot_rom_deg	-0.2331
pose_right_arm_swing_vel	-0.2079
pose_left_hip_angvel_mean	-0.0917
pose_left_foot_angvel_std	0.0845
pose_pelvis_angvel_mean	0.0647
pose_left_arm_swing_vel	0.0461
pose_spine3_angvel_std	0

Elastic-net logistic regression coefficients of the frozen model (standardized feature scale); 23 of 24 coefficients are non-zero. Signs are partial weights under collinearity and should not be read as marginal feature directions (see main text).

Supplementary Table 3 | Within-state reliability of PD-GaitState in CARE-PD (walk level, state-aware units)

Cohort	State unit	Units	Walks	Median walks/unit	ICC(1) [95% CI]	SEM (z)	MDC95 (z)
BMCLab	subject x med x rating	42	781	20	0.553 [0.402, 0.655]	1.29	3.575
E-LC	subject x med	74	160	2	0.69 [0.553, 0.838]	1.076	2.983
3DGait	subject x rating	25	56	2	0.047 [-0.185, 0.247]	0.892	2.472
PD-GaM	subject x rating	79	1701	20	0.71 [0.615, 0.778]	0.827	2.292
T-SDU-PD	subject x rating	21	379	17	0.383 [0.093, 0.567]	0.634	1.756
DNE (upper bound)	subject	95	474	5	0.466 [0.36, 0.563]	1.342	3.721

One-way random-effects variance components on walk-level scores; the state unit uses the finest available labels so that genuine state differences are not booked as measurement error. DNE carries no state label and its values are an upper bound on noise (residual state variation remains in the error term). Across the five state-labelled cohorts: SEM 0.634–1.29 z, MDC95 1.756–3.575 z, ICC(1) 0.047–0.71. Subject-mean MDC95 at k walks per state equals the walk-level MDC95 divided by √k (BMCLab: k(median) = 20 → MDC95 ≈ 0.8 z). Internal same-session repeats were too sparse for estimation (single scored recording per condition; two same-session pairs in one patient: |Δz| = 0.58 and 0.25). Because each local recording aggregates ≈5 walking passes before feature extraction while CARE-PD scores refer to single bouts, the applicable per-recording MDC95 is ≈ the walk-level MDC95 / √5 (0.785–1.599 z). ICC CIs are unit-level bootstrap percentiles (1,000 resamples). The within-state ICC does not bound the severity-anchor correlation: the anchor uses between-state and between-subject variance, which the within-state error term excludes by construction.

Supplementary Table 4 | Standardized response means (SRM) with 95% CIs for all paired treatment contrasts

Contrast	n pairs	Δ (z)	95% CI	P (paired t)	SRM	SRM 95% CI (bootstrap)
Internal Medication (DBS OFF)	50	1.195	[0.931, 1.458]	4.04e-12	1.29	[1.03, 1.66]
Internal Medication (DBS ON)	60	0.712	[0.449, 0.975]	1.15e-6	0.7	[0.43, 1.06]
Internal DBS (Med OFF)	52	0.725	[0.542, 0.909]	1.79e-10	1.1	[0.82, 1.46]
Internal DBS (Med ON)	55	0.334	[0.14, 0.529]	1.10e-3	0.47	[0.22, 0.75]
Prospective medication (pooled)	19	0.87	[0.511, 1.23]	7.73e-5	1.17	[0.75, 1.99]
Prospective DBS (pooled)	21	0.628	[0.404, 0.853]	1.04e-5	1.27	[0.91, 1.94]
External BMCLab medication	19	0.733	[0.348, 1.119]	8.48e-4	0.92	[0.6, 1.36]
External E-LC medication	17	1.238	[0.477, 2]	3.32e-3	0.84	[0.63, 1.4]

Internal contrasts use per-patient condition means of usable postoperative retrospective recordings (all patients with both conditions; larger n than the McNemar tables, which additionally require item 3.10 in both conditions). Prospective contrasts pool each patient's paired condition means across the concurrent therapy state. For symmetry, the SRM of the ordinal item-3.10 change on the identical pairs: 0.83 (internal medication, n = 28) and 0.54 (BMCLab, n = 19).

Supplementary Table 5 | Detection-threshold sensitivity analysis of the McNemar comparison and split-half specificity

Contrast	Improvement threshold (z)	PD-GaitState improved	Item 3.10 improved	Discordant (GS only / 3.10 only)	P (one-sided McNemar)
Medication (DBS OFF)	threshold 0.0	100%	67.9%	9 / 0	0.0020
Medication (DBS OFF)	threshold 0.25	96.4%	67.9%	8 / 0	0.0039
Medication (DBS OFF)	threshold 0.5	71.4%	67.9%	7 / 6	0.50
Medication (DBS OFF)	threshold 0.75	64.3%	67.9%	5 / 6	0.73
Medication (DBS OFF)	threshold 1.0	53.6%	67.9%	3 / 7	0.95
DBS (Med OFF)	threshold 0.0	90%	50%	13 / 1	0.00092
DBS (Med OFF)	threshold 0.25	80%	50%	11 / 2	0.011
DBS (Med OFF)	threshold 0.5	70%	50%	8 / 2	0.055
DBS (Med OFF)	threshold 0.75	53.3%	50%	4 / 3	0.50
DBS (Med OFF)	threshold 1.0	36.7%	50%	2 / 6	0.96
BMCLab medication (external)	threshold 0.0	84.2%	36.8%	9 / 0	0.0020
BMCLab medication (external)	threshold 0.25	73.7%	36.8%	8 / 1	0.020
BMCLab medication (external)	threshold 0.5	42.1%	36.8%	5 / 4	0.50
BMCLab medication (external)	threshold 0.75	36.8%	36.8%	5 / 5	0.62
BMCLab medication (external)	threshold 1.0	31.6%	36.8%	4 / 5	0.75
BMCLab medication (external)	threshold mdcmean_0.8	36.8%	36.8%	5 / 5	0.62

Split-half false-improvement rates under unchanged medication state (mean across 200 random splits): BMCLab: 50.0% at 0.0 z, 30.2% at 0.25 z, 16.4% at 0.5 z, 8.4% at 0.75 z, 4.4% at 1.0 z, 7.4% at 0.8 z; PD-GaM: 50.0% at 0.0 z, 19.7% at 0.25 z, 7.2% at 0.5 z, 2.6% at 0.75 z, 0.9% at 1.0 z; T-SDU-PD: 50.0% at 0.0 z, 14.7% at 0.25 z, 3.1% at 0.5 z, 0.9% at 0.75 z, 0.2% at 1.0 z. For symmetry, the modal BMCLab item-3.10 rating never changed under the same split-half protocol (false-change rate 0%, 42 subject-state units): the threshold-0 comparison therefore contrasts a continuous measure at 50% false-improvement with an ordinal measure at ≈0%, and the MDC-thresholded rows provide the specificity-matched comparison.

Supplementary Table 6 | Matched head-to-head comparison of treatment-state and severity supervision

Evaluation	Treatment-state supervised (PD-GaitState target)	Severity supervised (item 3.10 target)
Training basis (shared)	69 recordings, 36 patients	69 recordings, 36 patients

Clinical ratings required for training	0	69
Extreme-state discrimination, out-of-fold AUROC	0.880 [0.818, 0.941]	0.903 [0.829, 0.965]
Paired difference (state − severity), patient-level bootstrap	ΔAUROC −0.024 [−0.078, 0.035], P = 0.45	—
External 3DGait: subject-level ρ with the UPDRS gait item (n = 43)	−0.563 (P = 0.000085)	−0.592 (P = 0.000029)
External BMCLab: subject-level ρ with the UPDRS gait item (n = 23)	−0.38 (P = 0.073)	−0.529 (P = 0.0095)
External PD-GaM: subject-level ρ with the UPDRS gait item (n = 30)	−0.549 (P = 0.0017)	−0.681 (P = 0.000035)
External T-SDU-PD: subject-level ρ with the UPDRS gait item (n = 14)	−0.493 (P = 0.073)	−0.634 (P = 0.015)
External BMCLab: medication SRM (n = 19)	0.85 (P = 0.0016)	0.95 (P = 0.00063)
External E-LC: medication SRM (n = 17)	0.9 (P = 0.0019)	0.99 (P = 0.00085)

Both models are elastic nets on the same 24 features, trained on one common basis (the derivation recordings that also carry an item-3.10 rating) under a single shared patient-grouped fold structure, and compared on out-of-fold predictions for the same held-out recordings; the paired difference is bootstrapped over patients (2,000 resamples). Hyperparameters were tuned inside training folds (state model C = 0.05, l1 = 0.5; severity model α = 1, l1 = 0.1). The two supervision schemes are statistically indistinguishable on extreme-state discrimination, the task the biomarker is built for, while the severity-supervised model anchors the external UPDRS gait item slightly more strongly in all four labelled cohorts, as expected for a model trained on that rating. The 69-recording state model tabulated here is a like-for-like comparator and not the frozen PD-GaitState instrument, which was derived on the full 119 recordings from 53 patients; building a matched comparison costs 17 of those patients, because item 3.10 was unavailable for part of the cohort.

Supplementary Table 7 | Robustness of the frozen score to feature-level Gaussian noise (CARE-PD, 50 replicates per level)

Noise SD (× feature SD)	Score rank stability (ρ, median)	BMCLab Δ (z, median)	BMCLab P (median; % significant)	E-LC Δ (z, median)	E-LC P (median; % significant)	Pooled UPDRS ρ (median)
0.05	0.999	0.732	0.00086; 100%	1.238	0.0034; 100%	-0.501
0.1	0.996	0.732	0.00084; 100%	1.233	0.0035; 100%	-0.495
0.2	0.984	0.728	0.00088; 100%	1.242	0.0038; 100%	-0.491
0.3	0.965	0.726	0.0011; 100%	1.245	0.0045; 100%	-0.487
0.5	0.911	0.744	0.00085; 100%	1.264	0.0064; 100%	-0.462

Scorer re-implemented from persisted coefficients and scaling constants; validation against stored scores: Pearson r = 1 (internal) and 1 (CARE-PD). Baseline pooled subject-level UPDRS ρ = -0.503.

Supplementary Table 8 | Associations of PD-GaitState with axial MDS-UPDRS items and dyskinesia (internal cohort)

Item	Condition-level ρ (P)	Subject-level ρ (P)	Within-patient Δ ρ (P; n)
Item 3.11 (freezing of gait)	-0.349 (0.000028)	-0.138 (0.36)	-0.806 (2.3e-7; n = 28)
Item 3.12 (postural stability)	-0.426 (2.0e-8)	-0.631 (0.0000020)	-0.173 (0.33; n = 34)
Axial composite (3.1, 3.9–3.13)	-0.525 (7.6e-13)	-0.575 (0.000024)	-0.607 (0.00011; n = 35)

Subject-clustered condition-level inference (OLS, cluster-robust SE): item 3.11 β = -0.348 [-0.537, -0.158], P = 0.00032; item 3.12 β = -0.649 [-0.88, -0.419], P = 3.4e-8; axial composite β = -2.223 [-2.847, -1.599], P = 2.9e-12 (points per z). Condition-matched items from the clinical database; visit-condition mapping verified with 100% agreement against the item 3.10 lookup. Dyskinesia check: 46 patients with ON-state item 4.1 ratings, 15 with any dyskinesia; ON-state score vs item 4.1: ρ = 0.185 (P = 0.22); dyskinetic vs non-dyskinetic median ON-state score 0.696 vs 0.496 (Mann-Whitney P = 0.33).

Supplementary Table 9 | Null references for the feature-monotonicity count

Axis	Replicates	Strictly monotone features (median of 23)	95th percentile	P(count ≥ observed 22)
PD-GaitState (observed)	—	22	—	—
Random unit axes (standardized feature space)	1,000	16	23	0.148
Elastic nets on within-patient permuted treatment labels	200	2	14	0

Same 273 postoperative runs and same strict-monotonicity criterion (all four adjacent quintile transitions in one direction) as the manuscript analysis. The permuted-label null shows per-feature monotonicity is not entailed by examining constituents of a fitted score; the random-axis null shows the correlated feature space partly favours monotone profiles. The analysis is accordingly presented as a descriptive characterization: it excludes two-cluster, cliff-like score behaviour but does not establish interval-scale properties.

Supplementary Table 10 | Single features versus the composite across the full validation ladder

Measure	Internal AUROC	BMCLab ρ	3DGait ρ	PD-GaM ρ	T-SDU-PD ρ	BMCLab med SRM (P)	E-LC med SRM (P)	Prosp. DB (P)
Head angular velocity	0.864 ± 0.045	-0.485	-0.433	-0.413	-0.727	0.75 (0.0043)	0.97 (0.0010)	+0.48 SD (0.050)
Right-shoulder ang. vel.	0.859 ± 0.093	-0.475	-0.516	-0.409	-0.52	0.65 (0.012)	0.69 (0.012)	+0.43 SD (0.094)
Left-shoulder ang. vel.	0.859 ± 0.067	-0.498	-0.516	-0.534	-0.077	0.8 (0.0026)	0.82 (0.0039)	+0.34 SD (
Upper-spine ang. vel.	0.858 ± 0.043	-0.552	-0.715	-0.67	-0.542	0.88 (0.0012)	0.83 (0.0035)	+0.37 SD (0.091)
Walking speed	0.712 ± 0.046	-0.838	-0.726	-0.656	-0.681	0.99 (0.00041)	0.99 (0.00085)	+0.11 m/s (0.21)*
PD-GaitState (composite)	0.877 (nested)	-0.464	-0.553	-0.605	-0.767	0.92 (0.00085)	0.84 (0.0033)	+0.70 z (3.

*Internal AUROC from the single-level patient-grouped benchmark (composite row shows the nested-CV value); subject-level Spearman ρ with the UPDRS gait item per labelled cohort; paired medication effects (SRM, paired t-test P); prospective DBS effect from per-feature mixed models on the 79 prospective recordings, in units of the feature's derivation SD (*walking speed row quotes the main-text model in m/s). No single feature is strong on every test: head/shoulder velocities weaken on external anchoring, upper-spine velocity misses the prospective DBS axis (P = 0.091), head angular velocity reaches it only marginally (P = 0.0496), and walking speed misses it entirely; the composite is significant on all tests.*
